# Improved PIEZO1 agonism through 4‐benzoic acid modification of Yoda1

**DOI:** 10.1111/bph.15996

**Published:** 2023-01-31

**Authors:** Gregory Parsonage, Kevin Cuthbertson, Naima Endesh, Nicoletta Murciano, Adam J. Hyman, Charlotte H. Revill, Oleksandr V. Povstyan, Eulashini Chuntharpursat‐Bon, Marjolaine Debant, Melanie J. Ludlow, Timothy Simon Futers, Laeticia Lichtenstein, Jacob A. Kinsella, Fiona Bartoli, Maria Giustina Rotordam, Nadine Becker, Andrea Brüggemann, Richard Foster, David J. Beech

**Affiliations:** ^1^ Leeds Institute of Cardiovascular and Metabolic Medicine, School of Medicine University of Leeds Leeds UK; ^2^ School of Chemistry University of Leeds Leeds UK; ^3^ Nanion Technologies GmbH Munich Germany; ^4^ Theoretical Medicine and Biosciences Saarland University Homburg Germany

**Keywords:** calcium channel, endothelial cell, mechanical force, medicinal chemistry, non‐selective cation channel, pharmacology, vascular biology

## Abstract

**Background and Purpose:**

The protein PIEZO1 forms mechanically activated, calcium‐permeable, non‐selective cation channels in numerous cell types from several species. Options for pharmacological modulation are limited and so we modified a small‐molecule agonist at PIEZO1 channels (Yoda1) to increase the ability to modulate these channels.

**Experimental Approach:**

Medicinal chemistry generated Yoda1 analogues that were tested in intracellular calcium and patch‐clamp assays on cultured cells exogenously expressing human or mouse PIEZO1 or mouse PIEZO2. Physicochemical assays and wire myography assays on veins from mice with genetic disruption of PIEZO1.

**Key Results:**

A Yoda1 analogue (KC159) containing 4‐benzoic acid instead of the pyrazine of Yoda1 and its potassium salt (KC289) have equivalent or improved reliability, efficacy and potency, compared with Yoda1 in functional assays. Tested against overexpressed mouse PIEZO1 in calcium assays, the order of potency (as EC_50_ values, nM) was KC289, 150 > KC159 280 > Yoda1, 600). These compounds were selective for PIEZO1 over other membrane proteins, and the physicochemical properties were more suited to physiological conditions than those of Yoda1. The vasorelaxant effects were consistent with PIEZO1 agonism. In contrast, substitution with 2‐benzoic acid failed to generate a modulator.

**Conclusion and Implications:**

4‐Benzoic acid modification of Yoda1 improves PIEZO1 agonist activity at PIEZO1 channels. We suggest naming this new modulator Yoda2. It should be a useful tool compound in physiological assays and facilitate efforts to identify a binding site. Such compounds may have therapeutic potential, for example, in diseases linked genetically to PIEZO1 such as lymphatic dysplasia.

## Graphical abstract

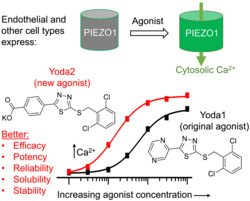

**Abbreviations**
ECGM‐2: endothelial cell basal medium 2
GFP: green fluorescent protein
hPIEZO1: human PIEZO1
HUVECs: human umbilical vein endothelial cells
mPIEZO1: mouse PIEZO1
mPIEZO2: mouse PIEZO2
M‐Stim: mechanical stimulation
PTFE: polytetrafluoroethylene
RED: rapid equilibrium dialysis
siCtrl: control short‐interfering RNA
siPIEZO1: short‐interfering RNA targeted to PIEZO1 expression
siPIEZO2: short‐interfering RNA targeted to PIEZO2 expression

**What is already known**

- PIEZO1 channels are important sensors of mechanical force.
- Options for pharmacological modulation of PIEZO1 are limited.

**What does this study add**

- Data on novel PIEZO1 channel agonists and new agonist structure–activity relationships.
- New compounds with improved agonist activity at PIEZO1 channels.

**What is the clinical significance**

- Potential foundations for treating diseases linked genetically to PIEZO1, such as lymphatic dysplasia.

## 1 INTRODUCTION

The protein PIEZO1 is a mechanically activated calcium ion (Ca^2+^)‐permeable non‐selective cation channel subunit (Coste et al., 2010; Murthy et al., 2017). It assembles as trimers to form a central ion pore (Guo & MacKinnon, 2017; Jiang et al., 2021). Its N‐terminal propeller blade‐like structures curve and indent the membrane, most likely to enable response to increased membrane tension (De Vecchis et al., 2021; Guo & MacKinnon, 2017; Yang et al., 2022; Young et al., 2022). Channel activation leads to Ca^2+^ influx and intracellular Ca^2+^ elevation and thus cellular effects due to the second messenger roles of Ca^2+^. The channels are widely expressed and have diverse functions, including in blood flow sensing by endothelium (Li et al., 2014; Rode et al., 2017; Wang et al., 2016) and detection of cyclical force in innate immunity (Solis et al., 2019). Mutations in human *PIEZO1* (hPIEZO1) are associated with anaemia (Zarychanski et al., 2012), malarial resistance (Ma et al., 2018), lymphatic dysplasia (Fotiou et al., 2015) and varicose vein disease (Fukaya et al., 2018), suggesting important roles in red blood cell, vascular wall and other aspects of human biology (Beech & Kalli, 2019; Jiang et al., 2021).

The pharmacology available for PIEZO1 is limited, and so the opportunities for PIEZO1 manipulation and therapeutic drug discovery are restricted. Most importantly so far, screening of a library of small molecules revealed a promising agent called Yoda1, named in reference to the ‘may the force be with you’ phrase of the Star Wars films (Syeda et al., 2015). This substance (2‐[5‐[[(2,6‐dichlorophenyl)methyl]thio]‐1,3,4‐thiadiazol‐2‐yl]pyrazine) is an agonist of mouse and human PIEZO1 (hPIEZO) channels (Syeda et al., 2015). It does not activate the only related channel, PIEZO2, in studies of mouse PIEZO2 (mPIEZO2) overexpressed in HEK 293 cells (Syeda et al., 2015). Yoda1 is used extensively in the field and, although some concern has been raised about its suitability, it appears to be a valuable tool compound (Beech & Kalli, 2019). The compound activates PIEZO1 channels reconstituted in lipid bilayers (Syeda et al., 2015) and may act somewhere in the propeller blade region proximal to the ion pore domain, putatively serving as a ‘molecular wedge’ to lower the threshold for mechanical activation (Botello‐Smith et al., 2019). Despite the synergy with mechanical force (Syeda et al., 2015), direct application of Yoda1 in the absence of exogenous force is often sufficient to cause strong activation of PIEZO1 channels (Evans et al., 2018), perhaps because there is already endogenous force acting on the channel (e.g., from the substrate or cell–cell contact). Therefore, Yoda1 can be used as a test agent to determine the presence of functional PIEZO1 channels and indicate physiological roles of PIEZO1. Nevertheless, although Yoda1 is a useful tool compound, it has limitations such as its relatively low potency and aqueous solubility (Syeda et al., 2015).

A potential route to improving PIEZO1 pharmacology is through better understanding of the structure–activity relationships of Yoda1. Integrity of the 2,6‐dichlorophenyl moiety is important (Evans et al., 2018; Syeda et al., 2015) with only limited possibilities for modification with retained activity (Li et al., 2021). The pyrazine moiety at the other end of the molecule, however, appears to be less critical while still important for agonism (Evans et al., 2018). Therefore, we hypothesised that modification of the pyrazine could be a route to improved agonism and physicochemical properties.

## 2 METHODS

### 2.1 Stable cell lines

HEK T‐REx™ 293 cells (Thermo Fisher Cat #R71007, RRID:CVCL_D585) that overexpress hPIEZO1 upon induction with tetracycline were generated as previously described (Rode et al., 2017). PIEZO1‐GFP was used as a PCR template to clone hPIEZO1 coding sequence into pcDNA™4/TO between HindIII and EcoRI restriction sites. PIEZO1 was amplified as two fragments using the following primers: (HindIII‐PIEZO1‐Fw: AATAAGCTTATGGAGCCGCACGTG and BamHI‐Int.PIEZO1‐Rv: AATGGATCCCCCTGGACTGTCG) and (BamHI‐Int. PIEZO1‐Fw: AATGGATCCTCCCCGCCACGGA and EcoRI‐PIEZO1‐Rv: AATGAATTCTTACTCCTTCTCACGAGT). The two fragments were fused using BamHI restriction site, resulting in the full‐length PIEZO1 coding sequence with the c4182a silent mutation. HEK T‐REx™ 293 cells (Invitrogen) were transfected with pcDNA4/TO‐PIEZO1 using Lipofectamine 2000 (Thermo Fisher Scientific). Subsequently, cells were treated with 10 μg·ml^−1^ blasticidin and 200 μg·ml^−1^ zeocin (InvivoGen) to select stably transfected cells. Single‐cell clones were isolated and analysed individually. Expression was induced by treating the cells for 24 h with 10 ng·ml^−1^ tetracycline and analysed by quantitative RT‐PCR and Western blot. These cells are referred to as hPIEZO1‐TREx.

HEK T‐REx™ cells that constitutively overexpress murine PIEZO1 were generated as previously described (Blythe et al., 2019). pcDNA3 mouse PIEZO1 (mPIEZO1) IRES green fluorescent protein (GFP), a gift from Ardem Patapoutian (Coste et al., 2010), was used as a template to clone the mPIEZO1 coding sequence into pcDNA4/TO. Overlapping mPIEZO1 (forward primer 5′‐GTAACAACTCCGCCCCATTG‐3′ and reverse primer 5′‐GCTTCTACTCCCTCTCACGTGTC‐3′) and pcDNA4/TO (forward primer 5′‐GACACGTGAGAGGGAGTAGAAGCCGCTGATCAGCCTCGACTG‐3′ and reverse primer 5′‐CAATGGGGCGGAGTTGTTAC‐3′) PCR products were assembled using Gibson Assembly (New England Biolabs). This construct does not contain tetracycline operator sequences. HEK T‐REx™ 293 cells were transfected with pcDNA4/TO‐mPIEZO1 using Lipofectamine 2000 (Invitrogen) and treated with 200 μg·ml^−1^ zeocin to select stably transfected cells. Individual clones were isolated and analysed for expression using Yoda1 and intracellular Ca^2+^ measurements. These cells are referred to as mPIEZO1‐TREx.

HEK T‐REx™ 293 cells stably expressing tetracycline‐regulated human TRPC5 have been described previously (Zeng et al., 2004). For the TRPC5‐expressing cells, selection was achieved by including 5 μg·ml^−1^ blasticidin and 400 μg·ml^−1^ zeocin in the cell medium. To induce expression, cells were incubated with 1 μg·ml^−1^ tetracycline for 24 h prior to experiments. These cells are referred to as hTRPC5‐TREx.

### 2.2 PIEZO2 and cell transfection

mPIEZO2 pCMV‐Sport6 was a gift from Ardem Patapoutian (Coste et al., 2015), obtained via Addgene (Addgene plasmid #81073; http://n2t.net/addgene:81073; RRID:Addgene_81073). It was sequenced for validation. Transient transfection of the plasmid was achieved using Lipofectamine 3000 in HEK 293 cells, 48 h prior to measurements being made.

### 2.3 Cell culture

HEK T‐REx™ 293 cells were maintained in DMEM, supplemented with 10% heat‐inactivated fetal calf serum, 100 U·ml^−1^ penicillin and 100 μg·ml^−1^ streptomycin (Sigma‐Aldrich). Stably transfected HEK T‐REx™ 293 cells continued to receive 10 μg·ml^−1^ blasticidin and 200 μg·ml^−1^ zeocin in maintenance cultivation. Non‐transfected HEK T‐REx™ 293 cells were used as control cells.

Human umbilical vein endothelial cells (HUVECs) purchased from PromoCell (#C‐12203) were maintained in endothelial cell basal medium 2 (ECGM‐2, PromoCell #C‐22111) supplemented with 500 pg·ml^−1^ recombinant human vascular endothelial growth factor 165, 10 ng·ml^−1^ recombinant human basic fibroblast growth factor, 200 ng·ml^−1^ hydrocortisone, 22.5 μg·ml^−1^ heparin and 2% (vol./vol.) fetal calf serum. Antibiotic–antimycotic (Gibco #15240062) was also added and resulted in final concentrations of 10 U·ml^−1^ penicillin, 10 U·ml^−1^ streptomycin and 250 ng·ml^−1^ amphotericin B. HUVECs were passaged at a maximum seeding ratio of 1:4 (parent:daughter flask) after 3 to 6 days' growth, when they had reached densities of between 3.4 and 6.6 × 10^4^ cells·cm^−2^. After washing the cell monolayer with D‐PBS (Sigma #D8537), 2 ml pre‐warmed 0.05% trypsin– EDTA (Gibco #25300054) was added to 75 cm^2^ flasks until the cells had detached (between 2 and 5 min at 37°C). Complete ECGM‐2 (8 ml) was added to inhibit the trypsin, the cells were counted and their density was adjusted before seeding into fresh vessels for maintenance cultivation or experimental purposes. The medium for maintenance of cultures was completely refreshed every 48 h. HUVECs were used for experiments after a minimum of three and a maximum of five passages. All cells were grown at 37°C and in 5% CO_2_ humidified incubator.

Hela (ATCC Cat #CRM‐CCL‐2, RRID:CVCL_0030) and HEK 293 (ATCC Cat #CRL‐1573, RRID:CVCL_0045) cells were purchased from ATCC and maintained in DMEM supplemented with 10% heat‐inactivated fetal calf serum, 100 U·ml^−1^ penicillin and 100 μg·ml^−1^ streptomycin (Sigma‐Aldrich).

### 2.4 RNA interference

Cells were transfected using Opti‐MEM™ I Reduced Serum Medium (Thermo Fisher Scientific) and Lipofectamine 2000 (Thermo Fisher Scientific). For transfection of cells in six‐well plates, 20 nmol short‐interfering RNA (siRNA) (control siRNA [siCtrl]: Dharmacon ON‐TARGETplus Non‐targeting Control Pool, D‐001810‐10‐05; siRNA targeted to PIEZO2 expression [siPIEZO2]: Dharmacon ON‐TARGETplus SmartPool PIEZO2, L‐013925‐02‐0005; and PIEZO1: Sigma‐Aldrich, GCAAGUUCGUGCGCGGAUU[DT][DT]) and 3 μl of Lipofectamine 2000 complexed in 200 μl of Opti‐MEM were added to 0.8 ml cell culture medium per well. The culture medium was changed 4 h post transfection, and cells were used for experiments 48 h later.

#### 2.4.1 RNA isolation and RT‐qPCR

RNA was isolated using TRIzol according to the manufacturer's protocol; 1 μg of total RNA was reverse transcribed using the iScript™ cDNA Synthesis Kit (Bio‐Rad), according to the manufacturer's instructions. qPCR was performed using SYBR Green (Bio‐Rad). qPCR reactions were performed on a LightCycler® 480 Real Time PCR System (Roche). Samples were analysed using the comparative CT method and expressed as percentage of glyceraldehyde 3‐phosphate dehydrogenase (GAPDH) housekeeping gene. The sequences of PCR primers (synthesised by Integrated DNA Technologies) were as follows:

**Table:** 

Species	Gene	Forward (5′–3′)	Reverse (5′–3′)
*Homo sapiens*	*PIEZO1*	CGTCTTCGTGGAGCAGATG	GCCCTTGACGGTGCATAC
	*PIEZO2*	GACAGACGAAGCAGCACAGA	GTGCTTTCTTCCAACTCGCC
	*GAPDH*	GCCTCAAGATCATCAGCAAT	GGACTGTGGTCATGAGTCCT
*Mus musculus*	*Piezo1*	TGAGCCCTTCCCCAACAATAC	CTGCAGGTGGTTCTGGATATAG
	*Piezo2*	AGAGTCGGAAAAGAGATACCCTC	CCAGACGATACAGATGAGAAGGA

### 2.5 Ca^2+^ measurement

HEK 293‐TREx cells and their stably transfected derivatives or transiently transfected HEK 293 or HeLa cells were seeded at a density of 7.5 × 10^4^ cells per well into poly‐d‐lysine‐coated 96‐well plates. The plates were either commercially pre‐coated with poly‐d‐lysine (Greiner Bio‐One #655946) or (Greiner Bio‐One #655090) were coated in‐house with 35 μl of a 10 μg·ml^−1^ poly‐d‐lysine (Bio‐Techne, Cultrex # 3439‐100‐01) in sterile deionised water per well for at least 24 h and rinsed three times with 100 μl per well sterile D‐PBS before addition of cells. HUVECs were seeded into tissue culture‐treated 96‐well plates (Greiner Bio‐One #655090) at a density of either 2.5 × 10^4^ cells 24 h before experimentation or 1.25 × 10^4^ cells 48 h before experimentation. Cells were incubated with 2 μM Fura‐2‐AM (Molecular Probes™) in the presence of 0.01% (wt./vol.) pluronic F127 (Sigma‐Aldrich #P2443) in standard bath solution (SBS) for 1 h at 37°C. Cells were washed with SBS for 30 min at room temperature. Alternatively, if inhibitors were being tested, these were added at this time, immediately following a wash in SBS. (−)‐Englerin A (Akbulut et al., 2015) was used in SBS containing 0.01% pluronic acid as a dispersing agent to minimise aggregation of compound. Unless otherwise stated, the final concentration of DMSO was 0.2% (vol./vol). Working 2000× stock solutions of test compounds were freshly made in DMSO and diluted 1000‐fold in SBS during the 30 min SBS wash/inhibitor pre‐incubation period to obtain 2× solutions for injection. When testing the highest concentration of Yoda1, KC159 and KC289 (30 μM), the final DMSO concentration was 0.6% (vol./vol.). Measurements were made at room temperature on a 96‐well fluorescence plate reader (FlexStation 3, Molecular Devices, Sunnyvale, CA, USA) controlled by SoftMax Pro software v7.0.3. Recipient wells containing 80 μl SBS/0.2% (vol./vol.) DMSO vehicle or inhibitor compound received 80 μl 2× compound at an injection rate of 1 μl·s^−1^, and the pipette introduced liquids from a height setting of 70 μl. No trituration was performed. For recordings using Fura‐2, the change in intracellular Ca was indicated as the ratio of Fura‐2 emission (510 nm) intensities at 340 and 380 nm excitation. Photomultipliers were set to medium sensitivity. SBS contained 130 mM NaCl, 5 mM KCl, 8 mM d‐glucose, 10 mM HEPES, 1.2 mM MgCl_2_ and 1.5 mM CaCl_2_, and the pH was adjusted to 7.4 with NaOH. The solution was filtered through a 0.2 μm bottle‐top filter prior to use and handled aseptically thereafter.

We did not randomise compounds in the multiwell plate Ca^2+^ assays because of the risk of it introducing errors due to the complexity of constructing and analysing randomised plates. We did, however, take steps to minimise the risk of systematic errors by altering the position of specific compounds in the plate to test for the possibility of position‐related effects, for which we found no evidence.

### 2.6 Patch‐clamp recording

For manual patch clamp, macroscopic transmembrane ionic currents of mPIEZO1 HEK‐TREx cells (or control HEK‐TREx cells) were recorded using the standard whole‐cell configuration for patch clamp in voltage‐clamp mode. For studies of HEK 293 cells overexpressing PIEZO2 (or control HEK 293 cells), the outside‐out patch configuration was used in voltage‐clamp mode. All recordings were made using an Axopatch 200B amplifier (Axon Instruments, Inc., USA) equipped with Digidata 1550B hosted by a PC running pClamp 10.7 software (Molecular Devices, USA) at room temperature. The cells were maintained during the experiment in an external salt buffer solution (SBS) of the following composition: 135 mM NaCl, 5 mM KCl, 1.5 mM CaCl_2_, 1.2 mM MgCl_2_, 8 mM d‐glucose and 10 mM HEPES (titrated to pH 7.4 with NaOH). Patch pipettes were fire‐polished and had a resistance of 4–7 MΩ when filled with the pipette solution of the following composition: 145 mM CsCl, 2 mM MgCl_2_, 10 mM HEPES, 1 mM EGTA, 5 mM Na_2_ATP and 0.1 mM Na_2_GTP, and the pH was titrated to 7.2 with CsOH (whole‐cell experiments). For outside‐out experiments, Na_2_ATP and Na_2_GTP were omitted. For whole‐cell experiments, cells were held at 0 mV and current amplitude was monitored by application of ramp voltage protocol from −100 to +100 mV at 10 s intervals. Currents were measured at −100 and +100 mV. Test compounds were applied in the external solution using a bath perfusion system. SBS flow was applied to the cells for 1 min before the addition of compounds. For outside‐out experiments, the patches were held at −80 mV and currents were activated by application of 100 ms pressure pulse from 0 to 75 mmHg applied directly to the patch pipette with an interval of 12.387 s using High Speed Pressure Clamp HSPC‐1 System (ALA Scientific Instruments, USA). Current records were analogue filtered at 1 kHz and digitally acquired at 10 kHz. Data were analysed and plotted using pClamp 10.7 and Microcal Origin 2018 (Microcal Software, USA).

For automated patch clamp, cells were cultured and harvested according to Nanion's standard cell culture protocol (Obergrussberger et al., 2014). hPIEZO1‐TREx cells were induced to express PIEZO1 by incubation with 0.5 μg·ml^−1^ tetracycline 24 h prior to experiments. Whole‐cell patch‐clamp recordings were conducted on HEK‐TREx, hPIEZO1‐TREx and mPIEZO1‐TREx cells according to Nanion's standard procedure for the SyncroPatch 384, using medium resistance (4–5 MΩ) chips (Obergrussberger et al., 2018). Cells were held at −80 mV for the duration of the experiment. The channel was activated by mechanical forces in the absence and presence of the agonists. To stimulate PIEZO1 channels mechanically (mechanical stimulation [M‐Stim]), we dispensed 20 μl of solution (either reference or agonist) locally to the cell at a pipetting speed of 110 μl·s^−1^ in synchrony with a triggered recording of the current response at the holding potential. The internal solution contained 110 mM KF, 10 mM KCl, 10 mM NaCl, 10 mM HEPES and 10 mM EGTA, pH adjusted to 7.2 with KOH, and the external solution contained 140 mM NaCl, 4 mM KCl, 2 mM CaCl_2_, 1 mM MgCl_2_, 5 mM glucose, 10 mM HEPES, pH adjusted to 7.4 with NaOH, and 0.1% DMSO to match the DMSO concentration of the compound solutions. For PIEZO1 stimulation, KC159, KC157 or Yoda1 was applied directly onto the cells via a pipette application. One chip of the SyncroPatch 384 is equipped with 384 wells served by 384 robotic pipettes and connected to 384 individual amplifiers, allowing us to test 384 distinct cells in one experiment. We tested the effect of each specified analogue on mPIEZO1‐, hPIEZO1‐ and untransfected HEK‐TREx cells in parallel and in comparison to mechanical and Yoda1 stimulation. To estimate the EC_50_ values, four concentrations of each agonist were tested per cell line in one chip, with each cell receiving single concentrations, and the concentration–response curve was calculated across the whole chip (384 cells). Only cells with seal resistance >0.3 GΩ, series resistance <20 MΩ and cell capacitance >5 and <40 pF were used for analysis. We considered responding cells as the cells treated with the agonist that displayed current with peak amplitude >−100 pA and area under the curve (AUC) >−10 pA.s.

### 2.7 Chemical synthesis

Analogues of Yoda1 were synthesised using a four‐step synthetic route utilising a Suzuki coupling to introduce diversity (Scheme 1), whereas compound KC289 is the potassium salt of KC159, synthesised by treating KC159 with KOH. All synthesised chemicals were purified by column chromatography or trituration and determined as >97% pure by ^1^H NMR and ^13^C NMR. Synthetic and analytical details are reported in the supporting information.

### 2.8 Eurofins' Hit Profiling Screen PP70

These experiments were performed by Eurofins Scientific, and the methods below are from this company's information. Membrane protein/membrane amounts may have varied, and the concentrations used were adjusted as necessary. KC289 was tested at 5 μM.

#### 2.8.1 Acetylcholine M_2_ receptors

Human recombinant muscarinic M_2_ receptors expressed in CHO‐K1 cells were used in modified Tris–HCl buffer pH 7.4. An 8 μg aliquot was incubated with 0.8 nM [^3^H]N‐methylscopolamine for 120 min at 25°C. Non‐specific binding was estimated in the presence of 1 μM atropine. Receptors were filtered and washed, and the filters were counted to determine [^3^H]*N*‐methylscopolamine specifically bound.

#### 2.8.2 Acetylcholine M_3_ receptors

Human recombinant muscarinic M_3_ receptors expressed in CHO‐K1 cells were used in modified Tris–HCl buffer pH 7.4. A 12 μg aliquot was incubated with 0.8 nM [^3^H]*N*‐methylscopolamine for 120 min at 25°C. Non‐specific binding was estimated in the presence of 1 μM atropine. Receptors were filtered and washed, and the filters were then counted to determine [^3^H]*N*‐methylscopolamine specifically bound.

#### 2.8.3 Adenosine A_1_ receptors

This assay measured binding of [^3^H]DPCPX to adenosine A_1_ receptors. CHO‐K1 cells stably transfected with a plasmid encoding the human adenosine A_1_ receptor were used to prepare membranes in modified HEPES pH 7.4 using standard techniques. A 10 μg aliquot of membrane was incubated with 1 nM [^3^H]DPCPX for 90 min at 25°C. Non‐specific binding was estimated in the presence of 100 μM R(−)‐PIA. Membranes were filtered and washed three times, and the filters were counted to determine [^3^H]DPCPX specifically bound.

#### 2.8.4 Adenosine A_2A_ receptors

This assay measured binding of [^3^H]CGS‐21680 to human adenosine A_2A_ receptors. HEK 293 cells stably transfected with a plasmid encoding the human adenosine A_2A_ receptor were used to prepare membranes in modified Tris–HCl pH 7.4 buffer using standard techniques. A 15 μg aliquot of membrane was incubated with 50 nM [^3^H]CGS‐21680 for 90 min at 25°C. Non‐specific binding was estimated in the presence of 50 μM NECA. Membranes were filtered and washed three times, and the filters were counted to determine [^3^H]CGS‐21680 specifically bound.

#### 2.8.5 α_1A_‐Adrenoceptors

Submaxillary glands of male Wistar‐derived rats weighing 175 ± 25 g were used to prepare α_1A_‐adrenoceptors in modified Tris–HCl buffer pH 7.4. A 5 mg aliquot was incubated with 0.25 nM [^3^H]prazosin for 60 min at 25°C. Non‐specific binding was estimated in the presence of 10 μM phentolamine. Membranes were filtered and washed, and the filters were then counted to determine [^3^H]prazosin specifically bound.

#### 2.8.6 α_1B_‐Adrenoceptors

The livers of male Wistar‐derived rats weighing 175 ± 25 g were used to prepare α_1B_‐adrenoceptors in modified Tris–HCl buffer pH 7.4. A 5 mg aliquot was incubated with 0.25 nM [^3^H]prazosin for 60 min at 25°C. Non‐specific binding was estimated in the presence of 10 μM phentolamine. Membranes were filtered and washed, and the filters were then counted to determine [^3^H]prazosin specifically bound.

#### 2.8.7 α_2A_‐Adrenoceptors

Human recombinant adrenergic α_2A_‐adrenoceptors expressed in CHO‐K1 cells were used in modified Tris–HCl buffer pH 7.4. A 2 μg aliquot was incubated with 1.5 nM [^3^H]rauwolscine for 60 min at 25°C. Non‐specific binding was estimated in the presence of 10 μM WB‐4101. Receptors were filtered and washed, and the filters were then counted to determine [^3^H]rauwolscine specifically bound. KC289 was screened at 5 μM.

#### 2.8.8 β_1_‐Adrenoceptors

Human recombinant β_1_‐adrenoceptors expressed in CHO‐K1 cells were used in modified Tris–HCl buffer pH 7.4. A 25 μg aliquot of membrane was incubated with 0.03 nM [^125^I]cyanopindolol for 120 min at 25°C. Non‐specific binding was estimated in the presence of 100 μM S(−)‐propranolol. Membranes were filtered and washed three times, and the filters were counted to determine [^125^I]cyanopindolol specifically bound.

#### 2.8.9 β_2_‐Adrenoceptors

This assay measured binding of [^3^H]CGP‐12177 to human β_2_‐adrenoceptors. Mammalian CHO‐hNBR1 cells stably transfected with a plasmid encoding the human β_2_‐adrenoceptor were used to prepare membranes in modified Tris–HCl pH 7.4 buffer using standard techniques. A 50 μg aliquot of membrane was incubated with 0.2 nM [^3^H]CGP‐12177 for 60 min at 25°C. Non‐specific binding was estimated in the presence of 10 μM ICI‐118551. Membranes were filtered and washed three times, and the filters were counted to determine [^3^H]CGP‐12177 specifically bound.

#### 2.8.10 Ca_v_1.2 (L type) channels

Cerebral cortices of Wistar‐derived rats weighing 175 ± 25 g were used to prepare membranes in Tris–HCl buffer pH 7.4. A 2.5 mg aliquot was incubated with 0.1 nM [^3^H]nitrendipine for 90 min at 25°C. Non‐specific binding was estimated in the presence of 1 μM nitrendipine. Membranes were filtered and washed, and the filters were then counted to determine [^3^H]nitrendipine specifically bound.

#### 2.8.11 Human CB_1_ receptors

Human recombinant cannabinoid CB_1_ receptors expressed in rat haematopoietic Chem‐1 cells were used in modified HEPES buffer pH 7.4. A 5 μg aliquot of membrane was incubated with 2 nM [^3^H]SR141716A for 60 min at 37°C. Non‐specific binding was estimated in the presence of 10 μM CP 55,940. Membranes were filtered and washed four times, and the filters were counted to determine [^3^H]SR141716A specifically bound.

#### 2.8.12 Dopamine D_1_ receptors

This assay measured binding of [^3^H]SCH‐23390 to human dopamine D_1_ receptors. CHO cells stably transfected with a plasmid encoding the human D_1_ receptor were used to prepare membranes in modified Tris–HCl pH 7.4 buffer using standard techniques. A 20 μg aliquot of membrane was incubated with 1.4 nM [^3^H]SCH‐23390 for 120 min at 37°C. Non‐specific binding was estimated in the presence of 10 μM (+)‐butaclamol. Membranes were filtered and washed three times, and the filters were counted to determine [^3^H]SCH‐23390 specifically bound.

#### 2.8.13 Dopamine D_2B_ receptors

This assay measured binding of [^3^H]spiperone to human dopamine D_2B_ receptors. CHO cells stably transfected with a plasmid encoding the human dopamine D_2B_ receptor were used to prepare membranes in modified Tris–HCl pH 7.4 buffer using standard techniques. A 15 μg aliquot of membrane was incubated with 0.16 nM [^3^H]spiperone for 120 min at 25°C. Non‐specific binding was estimated in the presence of 10 μM haloperidol. Membranes were filtered and washed three times, and the filters were counted to determine [^3^H]spiperone specifically bound.

#### 2.8.14 GABA_A_ receptors (1)

Whole brains (except cerebellum) of male Wistar‐derived rats weighing 175 ± 25 g were used to prepare GABA_A_ agonist site receptors in Tris–HCl buffer pH 7.4. A 10 mg aliquot was incubated with 1 nM [^3^H]muscimol for 10 min at 4°C. Non‐specific binding was estimated in the presence of 0.1 μM muscimol. Membranes were filtered and washed, and the filters were then counted to determine [^3^H]muscimol specifically bound.

#### 2.8.15 GABA_A_ receptors (2)

Whole brains (except cerebellum) of male Wistar‐derived rats weighing 175 ± 25 g were used to prepare GABA_A_ central benzodiazepine membrane receptors in Na–K phosphate buffer pH 7.4. A 5 mg aliquot was incubated with 1 nM [ ^3^H]flunitrazepam for 60 min at 25°C. Non‐specific binding was estimated in the presence of 10 μM diazepam. Membranes were filtered and washed, and the filters were then counted to determine [^3^H]flunitrazepam specifically bound.

#### 2.8.16 Glutamate receptors

Cerebral cortices of Wistar‐derived rats weighing 175 ± 25 g were used to prepare glutamate NMDA phencyclidine receptors in Tris–HCl buffer pH 7.4. A 6.3 mg (see footnote 1) aliquot was incubated with 4 nM [^3^H]TCP for 45 min at 25°C. Non‐specific binding was estimated in the presence of 1 μM dizocilpine ((+)‐MK‐801). Membranes were filtered and washed, and the filters were then counted to determine [^3^H]TCP specifically bound.

#### 2.8.17 Histamine H_1_ receptors

Human recombinant histamine H_1_ receptors expressed in CHO‐K1 cells were used in modified Tris–HCl buffer pH 7.4. A 10 μg aliquot was incubated with 1.2 nM [^3^H]pyrilamine for 180 min at 25°C. Non‐specific binding was estimated in the presence of 1 μM pyrilamine. Receptor proteins were filtered and washed, and the filters were then counted to determine [^3^H]pyrilamine specifically bound.

#### 2.8.18 Imidazoline I_2_ receptors

Brains (except cerebella) of male Wistar‐derived rats weighing 175 ± 25 g were used to prepare imidazoline I_2_ receptors in modified Tris–HCl buffer pH 7.4. A 15 mg aliquot was incubated with 2 nM [^3^H]idazoxan for 30 min at 25°C. Non‐specific binding was estimated in the presence of 1 μM idazoxan. Membranes were filtered and washed, and the filters were then counted to determine [^3^H]idazoxan specifically bound.

#### 2.8.19 μ‐Opioid receptors

Human μ receptors expressed in CHO‐K1 cells were used in modified Tris–HCl buffer pH 7.4. An 11 μg aliquot was incubated with 0.6 nM [^3^H]diprenorphine for 60 min at 25°C. Non‐specific binding was estimated in the presence of 10 μM naloxone. Membranes were filtered and washed, and the filters were then counted to determine [^3^H]diprenorphine specifically bound.

#### 2.8.20 Nicotinic acetylcholine receptors containing the α1 subunit

Human α1‐nAChRs expressed in RD cells were used in 150 mM NaCl, 4 mM KCl and 2.3 mM CaCl_2_. An aliquot was incubated with 0.6 nM [^125^I]α‐bungarotoxin for 120 min at 25°C. Non‐specific binding was estimated in the presence of 1 μM α‐bungarotoxin. Membranes were filtered and washed, and the filters were then counted to determine [^125^I]α‐bungarotoxin specifically bound.

#### 2.8.21 Nicotinic acetylcholine receptors

Human nicotinic acetylcholine receptors expressed in IMR‐32 cells were used in 20 mM HEPES, pH 7.5, 150 mM NaCl, 1.5 mM KCl, 2 mM CaCl_2_ and 1 mM MgSO_4_. An aliquot was incubated with 0.10 nM [^125^I]epibatidine for 60 min at 25°C. Non‐specific binding was estimated in the presence of 300 μM (−)‐nicotine. Membranes were filtered and washed, and the filters were then counted to determine [^125^I]epibatidine specifically bound.

#### 2.8.22 Noradrenaline transporter

Human noradrenaline transporters expressed in dog kidney MDCK cells were used in modified Tris–HCl buffer pH 7.4. A 40 μg aliquot was incubated with 0.2 nM [^125^I]RTI‐55 for 3 h at 4°C. Non‐specific binding was estimated in the presence of 10 μM desipramine. Membranes were filtered and washed, and the filters were then counted to determine [^125^I]RTI‐55 specifically bound.

#### 2.8.23 Phosphodiesterases

This assay measured binding of [^3^H]rolipram to rolipram binding sites. Whole brains (except cerebellum) of male Wistar‐derived rats weighing 175 ± 25 g were prepared in modified Tris–HCl pH 7.4 buffer using standard techniques. A 180 μg aliquot of membrane was incubated with 1.8 nM [^3^H]rolipram for 60 min at 4°C. Non‐specific binding was estimated in the presence of 10 μM rolipram. Membranes were filtered and washed three times, and the filters were counted to determine [^3^H]rolipram specifically bound.

#### 2.8.24 Phorbol ester receptors

This assay measures binding of [^3^H]PDBu to phorbol ester receptors. Brain (except cerebella) membranes of male ICR‐derived mice weighing 20 ± 2 g were prepared in modified Tris–HCl pH 7.4 buffer using standard techniques. A 20 μg aliquot of membranes was incubated with 3 nM [^3^H]PDBu for 60 min at 25°C. Non‐specific binding was estimated in the presence of 1 μM PDBu. Membranes were filtered and washed three times, and the filters were counted to determine [^3^H]PDBu specifically bound.

#### 2.8.25 K_v_11.x (hERG) channels

Human recombinant hERG expressed in human HEK 293 cells was used in modified HEPES buffer pH 7.4. A 10 μg aliquot was incubated with 1.5 nM [^3^H]astemizole for 60 min at 25°C. Non‐specific binding was estimated in the presence of 10 μM astemizole. Channel proteins were filtered and washed, and the filters were then counted to determine [^3^H]astemizole specifically bound.

#### 2.8.26 K_ATP_ channels

This assay measured binding of [^3^H]glyburide to voltage‐insensitive, ATP‐sensitive, potassium channel sites (K_ATP_). HIT‐T15 Syrian hamster pancreatic beta cells were used to prepare membranes in modified MOPS pH 7.4 buffer using standard techniques. A 100 μg aliquot of membrane was incubated with 5 nM [^3^H]glyburide for 120 min at 25°C. Non‐specific binding was estimated in the presence of 1 μM glyburide. Membranes were filtered and washed three times, and the filters were counted to determine [^3^H]glyburide specifically bound.

#### 2.8.27 Prostanoid EP_4_ receptors

Human recombinant prostanoid EP_4_ receptors expressed in Chem‐1 cells were used in modified MES buffer pH 6.0. A 3 μg·ml^−1^ (see footnote 1) aliquot was incubated with 1 nM [^3^H]PGE_2_ for 120 min at 25°C. Non‐specific binding was estimated in the presence of 10 μM PGE_2_ . Receptors were filtered and washed, and the filters were then counted to determine [^3^H]PGE_2_ specifically bound.

#### 2.8.28 5‐HT_2B_ receptors

This assay measured binding of [^3^H]lysergic acid diethylamide (LSD) to human 5‐HT_2B_ receptors. CHO‐K1 cells stably transfected with a plasmid encoding the human 5‐HT_2B_ receptor were used to prepare membranes in modified Tris–HCl pH 7.4 buffer using standard techniques. A 30 μg aliquot of membrane protein was incubated with 1.2 nM [^3^H]LSD for 60 min at 37°C. Non‐specific binding was estimated in the presence of 10 μM 5‐HT. Membranes were filtered and washed three times, and the filters were counted to determine [^3^H]LSD specifically bound.

#### 2.8.29 Sigma 1 receptors

This assay measured binding of [^3^H]haloperidol to sigma 1 receptors. Human Jurkat cells were used to prepare membranes in potassium phosphate buffer pH 7.5 using standard techniques. A 140 μg aliquot of membrane was incubated with 8 nM [^3^H]haloperidol for 4 h at 25°C. Non‐specific binding was estimated in the presence of 10 μM haloperidol. Membranes were filtered and washed three times, and the filters were counted to determine [^3^H]haloperidol specifically bound.

#### 2.8.30 Na_v_ channels

This assay measured binding of [^3^H]batrachotoxin to site 2 of the sodium channel—batrachotoxin. Whole‐brain (except cerebellum) membranes of male Wistar‐derived rats weighing 175 ± 25 g were prepared in modified HEPES/Tris–HCl containing 40 mg·ml^−1^ LqTx (α scorpion toxin from *Leiurus quinquestriatus*) to block site 2 at pH 7.4 buffer using standard techniques. A 7.5 mg aliquot of membrane was incubated with 5 nM [^3^H]batrachotoxinin for 60 min at 37°C. Non‐specific binding was estimated in the presence of 100 μM veratridine. Membranes were filtered and washed three times, and the filters were counted to determine [^3^H]batrachotoxinin specifically bound.

### 2.9 Aqueous solubility

Standard curves for each test compound were constructed. DMSO‐dissolved test compounds were added to universal aqueous buffer (45 mM ethanolamine, 45 mM KH_2_PO_4_ and 45 mM KOAc, pH 7.4): acetonitrile 80:20 (298.5 μl) at a final DMSO concentration of 0.5%. Samples were incubated at room temperature with 300 rpm shaking for 30 min; 200 μl of each well was transferred to a 96‐well polypropylene V‐bottomed collection plate, and absorbance readings were taken at 250–500 nm in 10 nm increments. Thus, the *λ*_max_ was identified at which concentration versus absorbance was plotted to provide standard curves.

For estimating aqueous solubility, 2 μl of 50 mM test compound in DMSO was added to 398 μl of universal aqueous buffer and the samples were incubated for 1.5 h with 300 rpm shaking at room temperature. After filtration through hydrophilic PTFE 0.2 μm filters, 160 μl sample was transferred to wells of a 96‐well polypropylene V‐bottomed collection plate and 40 μl acetonitrile was added to each well. Absorbance readings were taken at 250–500 nm in 10 nm increments. Solubility was calculated at the appropriate *λ*_max_ with reference to the standard curve.

Kinetic and thermodynamic solubility assays were also performed by Malvern Panalytical (UK). Briefly, 1000 μl of 0.1 M phosphate buffer (pH 7.4) received either 25 μl of a 10 mM DMSO stock of test compound (for kinetic solubility assays) or 1 mg of test compound (for thermodynamic solubility assays). The mixtures were shaken on an orbital mixer to reach equilibrium for 1 or 24 h, respectively. The equilibrated solution was centrifuged, and the supernatant was removed to a fresh vial and then re‐centrifuged. High‐ and low‐dilution samples were prepared from the secondary supernatant and quantified by LC–MS/MS against a standard curve.

### 2.10 Microsomal half‐life

Compound half‐lives were measured by Malvern Panalytical (UK). Briefly, microsomal mouse liver microsomes (1 mg protein·ml^−1^) were pre‐incubated with NADPH cofactor solution at 37°C. Biotransformation was initiated by addition and mixing of 1 μM test compound in a final volume of 350 μl; 25 μl aliquots were removed at 5 min intervals for 35 min and quenched in 300 μl ice‐cold methanol containing internal standard. The protein in the samples was then precipitated by centrifugation at 4°C, and the supernatant was analysed by LC–MS/MS to quantify the test parent compound remaining at each time point.

### 2.11 Plasma protein binding

Mouse plasma protein binding was measured by rapid equilibrium dialysis (RED) performed by Malvern Panalytical (UK). Briefly, mouse plasma was warmed to 37°C for 10 min, and test compound stock solution was added to achieve a 5 μM solution; 500 μl of dialysis buffer was added to one side of the chamber of the device insert (Piercenet, Dialysis membrane MWCO 8000) housed within a heated Teflon block, and the incubation was initiated by the addition of 300 μl of the test compound protein solution to the opposite chamber. Equilibrium was reached by shaking the device on an orbital mixer at 37°C for 4 h; 50 μl aliquots from the buffer and the plasma chambers were transferred into separate wells of a deep‐well plate; 50 μl of plasma was added to the buffer samples, and 50 μl of buffer was added to the plasma samples; 300 μl of ice‐cold acetonitrile containing internal standard was added to precipitate the protein. Samples were then centrifuged (2700 × *g* at 4°C for 20 min) to pellet the protein, and the supernatant was analysed by LC–MS/MS to quantify the % input compound unbound to plasma protein with reference to a standard curve.

### 2.12 Stability in plasma

Pooled heparinised mouse plasma was warmed to 37°C for 10 min, mixed and cleared of aggregated protein by centrifugation. Aliquots of the clear supernatant were transferred into the assay plate. Following equilibration to 37°C, biotransformation was initiated by adding and mixing of 1 μM compound solution in a final incubation volume of 300 μl; 25 μl aliquots were removed at 0, 5, 15, 30, 60 and 120 min and quenched in 300 μl ice‐cold acetonitrile containing internal standard. After storing the samples at −20°C for a minimum of 4 h, the protein in the samples was precipitated by centrifugation at 4°C, and the supernatants were analysed by LC–MS/MS to quantify the % input test compound remaining.

### 2.13 Mice

All animal care and experimental procedures were authorised by the University of Leeds Animal Ethics Committee and the UK Home Office under the authority of the UK Home Office Project Licences P606320FB and PP8169223. Animal studies are reported in compliance with the ARRIVE guidelines (Percie du Sert et al., 2020) and with the recommendations made by the British Journal of Pharmacology (Lilley et al., 2020). The mouse was selected for animal ex vivo studies because it is the smallest known mammalian species that can be used to provide blood vessels suitable for myography and enable suitable genetic manipulation, which in this study was used to test the role of PIEZO1 channels. The portal vein was selected as the tissue to study because our pilot studies had shown that this blood vessel has a robust response to PIEZO1 channel agonists that is more reliable and larger than we have observed for other blood vessels.

All mice were housed in GM500 individually ventilated cages (Animal Care Systems) at 21°C and 50–70% humidity and with a 12 h alternating light/dark cycle. They had ad libitum access to water and RM1 diet (Special Diets Services, Witham, UK) with bedding from Pure‐o'Cel (Datesand, Manchester, UK). The number of cage companions was up to five. Animals were visually inspected and weighed at a minimum of weekly intervals for welfare‐related assessments. Local animal welfare advice and steps were taken in the rare cases of concern for an animal or animals. The genetically modified mice did not display any obvious adverse effects. Animals weighed 25–35 g.

Genotypes were determined by a service using real‐time PCR with specific probes designed for each gene (Transnetyx, Cordova, TN, USA). C57BL/6J mice from the University of Leeds with *PIEZO1* gene flanked with LoxP sites (PIEZO1^flox^) (Li et al., 2014) were used to generate tamoxifen‐inducible disruption of the *PIEZO1* gene in the endothelium. PIEZO1^flox/flox^ mice were crossed with mice expressing cre recombinase under the cadherin 5 promoter (Tg(Cdh5‐cre/ERT2)1Rha) and inbred to obtain PIEZO1^flox/flox^/Cdh5‐cre mice (Rode et al., 2017). Tamoxifen was dissolved in corn oil at 20 mg·ml^−1^. Mice were injected i.p. with 75 mg·kg^−1^ tamoxifen for five consecutive days, and studies were performed 10–14 days later. PIEZO1^flox/flox^/Cdh5‐cre mice that received tamoxifen injections are referred to as PIEZO1^ΔEC^. PIEZO1^flox/flox^ littermates (lacking Cdh5‐cre) that received tamoxifen injections were the controls (control genotype).

For experiments, the mice used were males aged 12–16 weeks. Only male mice were used in order to reduce variability that might arise due to sex differences and reproductive cycle. Tamoxifen injections and genotyping were performed by a researcher independently from the myographer, such that the genotypes were blind to the myographer. The different genotypes were studied at random as they became available, depending on the genotypic spread of each litter.

The study sought to address the 3Rs by using in vitro cell‐based (non‐animal) technical approaches as much as possible (i.e., Replacement), only using animals once the aim and design of such studies were informed by the in vitro studies (i.e., Reduction).

### 2.14 Myography

Mice were anaesthetised with isoflurane (5% induction and 1.5% maintenance) in 95% O_2_ according to Schedule 1 procedure approved by the UK Home Office. The portal vein was quickly dissected and placed in Krebs solution. The Krebs PV solution consisted of 118 mM NaCl, 4.7 mM KCl, 2.5 mM CaCl_2_, 1.2 mM KH_2_PO_4_, 1.2 mM MgSO_4_·7H_2_O, 25.2 mM NaHCO_3_ and 11.1 mM glucose. By means of a dissecting microscope, adhering perivascular tissue was carefully removed and the vein was cut into 1 mm segments.

Vein segments were mounted onto two thin stainless steel wires in an isometric myograph (Multi Wire Myograph System 620M from Danish Myograph Technology [DMT]), for which the force transducer was calibrated once per month according to the manufacturer's instructions. The recording chamber was filled with gassed Krebs solution (95% O_2_/5% CO_2_, pH 7.4). The segments were then stretched to a normalised internal diameter according to the manufacturer's instructions. The mounted rings were kept in a 5 ml chamber containing Krebs solution at 37°C and continuously bubbled with a gas mixture of 95% O_2_ and 5% CO_2_ (pH 7.4). After an equilibration period of 60 min, the contractile function of the vessel was tested by replacing the Krebs solution by 60 mM K^+^ solution. Following washout, the vein was contracted once with 10 μM phenylephrine and then Yoda1, KC159 or KC289 was applied with the concentrations of 0.1 to 10 μM. The NOS inhibitor, l‐NAME, (100 μM), was pre‐incubated for 20 min before the PIEZO1 modulator.

Because the anticipated effect of test compounds was to cause vessel relaxation, phenylephrine was applied to induce tension and the maximum value of this tension was defined as 0% relaxation (no relaxation). The value of tension immediately prior to application of phenylephrine was defined as 100% relaxation. Data are normalised to these values because, otherwise, the intrinsic variability in the absolute amount of tension induced by phenylephrine obscured exploration of the relaxant effect of compounds.

### 2.15 Data and statistical analysis

The identities of compounds and cell lines were not blinded to investigators. This is a limitation of the study that may have introduced bias. The data and statistical analysis in this study comply with the recommendations of the British Journal of Pharmacology on experimental design and analysis in pharmacology (Curtis et al., 2022). OriginPro 2020 (OriginLab) and Prism 9 (GraphPad) were used for all data analysis.

**FIGURE 1:**
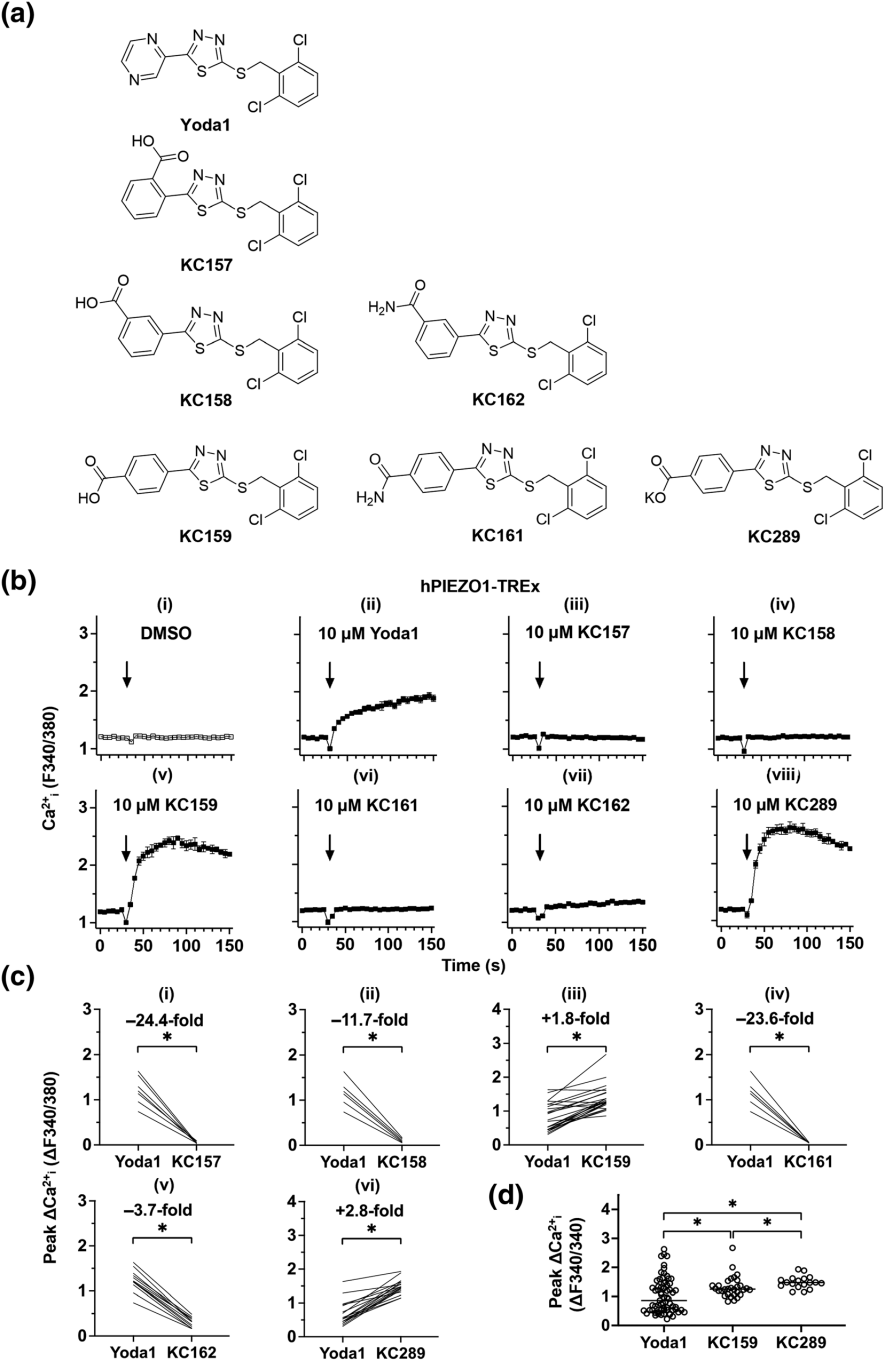
Yoda1 analogues and their effects on hPIEZO1. (a) Structures of Yoda1 and analogues in which the pyrazine moiety has been altered. KC157, 2‐benzoic acid; KC158, 3‐benzoic acid; KC159, 4‐benzoic acid; KC161, 4‐benzamide; KC162, 3‐benzamide; KC289, 4‐benzoic acid (potassium salt). (b) Intracellular Ca^2+^ measurements from a single experiment (n = 1) in which hPIEZO1‐TREx cells were acutely exposed to (i) DMSO vehicle or 10 μM (ii) Yoda1, (iii) KC157, (iv) KC158, (v) KC159, (vi) KC161, (vii) KC162 and (viii) KC289. Arrows indicate the time at which the indicated compound was added to the cells, following a 30 s background read. Mean (± SEM) values from three technical replicates are shown. (c) Paired, background‐subtracted (change above background, Δ), peak intracellular Ca^2+^ measurement comparisons of hPIEZO1‐TREx cells treated with 10 μM indicated compound. Each plot shows mean peak values from independent experiments (KC157, n = 7; KC158, n = 6; KC159, n = 25; KC161, n = 6; KC162, n = 12; and KC289, n = 18) and comparisons of the median values of the collated experiments are shown. ^*^ *P* < 0.05, significantly different as indicated; paired‐sample Wilcoxon signed‐rank test. Median fold differences compared to Yoda1 are also indicated. (d) Background‐subtracted (Δ) peak intracellular Ca^2+^ measurements for hPIEZO1‐TREx cells treated with 10 μM indicated compound. Each symbol shows a mean peak value from an independent experiment (Yoda1, n = 67; KC159, n = 30; and KC289, n = 18) and the median values of each treatment pair are shown. ^*^ *P* < 0.05, significantly different as indicated; Kruskal–Wallis ANOVA.

For intracellular Ca^2+^ (Ca^2+^i) measurements, technical intra‐experiment replicates were used to improve confidence in the data. Analysis of individual experiments was performed to obtain mean ± SEM values. For collated analysis of independent experiments, where normal distributions were not evidenced by Shapiro–Wilk normality tests, non‐parametric distributions were assumed, and therefore, median values were used. To compare the agonist activity of Yoda1 analogues, background readings taken for the first 25 s prior to compound injection were subtracted and the resulting median peak values were compared (ΔCa^2+^i). Data subjected to statistical analysis arose from at least five independent experiments (n = 5). For comparisons between two sets of paired data, Wilcoxon's signed‐rank tests were used. For comparisons of two sets of unpaired data, two‐tailed Mann–Whitney signed‐rank tests were performed. For multiple comparisons (Figure 1d), Kruskal–Wallis ANOVA was used. Datasets in Figures 10 and S10 (SI 1) satisfied the Shapiro–Wilk normality test, and therefore, repeated‐measure one‐way ANOVA was applied, followed by Tukey's post hoc test. Data for Figure S10 (SI 1A, ii) were not normally distributed, and so a Friedman test for paired non‐parametric data followed by Dunn's post hoc test was applied. *P* < 0.05 was deemed significant throughout. EC_50_ estimates from appropriately saturating concentration–response curves were fitted with a standard Hill equation (Hill1 function in OriginPro 2020 software).

DataControl 384 (Nanion Technologies GmbH), Prism 9 (GraphPad) and OriginPro 2020 (OriginLab) were used for all analysis of automated patch‐clamp data. For manual and automated patch‐clamp data, n represents the number of cells recorded from successfully in each experimental condition, sampled from two to six batches of cells or 384‐well chips in automated patch clamp. For manual patch‐clamp experiments, each cell/patch recording (n) is considered as an independent experiment because each was made independently at a separate time, using a separate coverslip of cells and using a separate patch pipette. In some instances, more than one such independent recording was made from the same batch of cultured or transfected cells. These n values are considered as independent. Each cell was studied alone and from a separate coverslip in the case for manual patch clamp. To estimate the EC_50_ values of the agonists in automated patch‐clamp studies, the peak current in the presence of the agonist was normalised to the peak current in the presence of the reference solution. Single‐point concentration–response curves were fitted with a standard Hill equation to estimate EC_50_ values. *P* < 0.05 was deemed significant. For automated patch‐clamp experiments, we included some data for n < 5 (Figure 7a,b). We did not apply a statistical test. We suggest that the results are, nevertheless, useful because of the high number of independent replicates per experiment (i.e., separate recordings from separate wells in each recording chip, which are referred to as n). Such replicates are a feature of, and a rationale for, this automated technology, which is an innovation for the type of research shown here but already commonly used in the pharmaceutical industry for other ion channel studies to increase throughput and avoid waste from large volumes of independent cell cultures and minimise cost from expensive specially engineered plates. As a general strategy, our study used multiple independent technical approaches to address the same or similar questions. These multiple approaches include Ca^2+^ measurement, patch clamp and vascular contraction assays. In this way, the overall conclusions do not rely on a single approach such as automated patch clamp.

OriginPro 2020 was used for the analysis of myography data. Myography traces show readings taken every 0.5 or 1 s and were smoothed with the Savitzky–Golay filter set to 70 points. To compare the agonistic activity of Yoda1 analogues, basal vessel tension prior to compound injection was subtracted. Maximal tension in the presence of PE alone was used to define 0% relaxation. The tension value prior to PE application was defined as 100% relaxation. Following the addition of test compounds, tension readings were taken at the last time point of the treatments and expressed as percentage relaxation.

Group sizes were not always equal because we did more experiments with analogues that were most active or newest. We have fully represented what we did rather than excluding data. Although our target was five, our observation of unexpected variability in effects of some compounds caused us to increase the number in some cases in an effort to increase the robustness of our conclusions. Statistical analysis was undertaken only for studies where each group size was at least n = 5. Group size is the number of independent values. Statistical analysis was done using these independent values (i.e., not treating technical replicates as independent values). For multigroup studies with parametric variables, post hoc tests were conducted only if *F* in ANOVA (or equivalent) achieved the chosen necessary level of statistical significance and there was no significant variance inhomogeneity. Potential outlier data points were retained in data analysis and presentation. A separate ‘Compliance with BJP Declaration of Transparency and Scientific Rigour’ document is provided. A separate data transparency spreadsheet document is provided containing all data.

### 2.16 Materials

Unless stated otherwise, all commercially available chemicals were purchased from Sigma‐Aldrich. Stocks of chemicals were reconstituted in DMSO and stored at −20°C unless stated otherwise. Fura‐2‐AM was dissolved at 1 mM. Pluronic acid F‐127 was stored at 10% (w/v) in DMSO at room temperature in the dark. Yoda1 (Tocris) was stored at 10 mM. All Yoda1 analogues were synthesised in‐house and purified (for more information, see the supporting information) and prepared as 10 mM stock solutions. (−)‐Englerin A was prepared as a 10 mM stock solution and stored at −80°C. PE was dissolved in distilled water to make a stock solution of 100 mM. l‐NAME was dissolved in distilled water to make a stock solution of 100 mM.

### 2.17 Nomenclature of targets and ligands

Key protein targets and ligands in this article are hyperlinked to corresponding entries in http://www.guidetopharmacology.org and are permanently archived in the Concise Guide to PHARMACOLOGY 2021/22 (Alexander, Christopoulos, et al., 2021; Alexander, Fabbro et al., 2021; Alexander, Kelly, et al., 2021; Alexander, Mathie et al., 2021).

## 3 RESULTS

### 3.1 4‐Benzoic acid substitution in Yoda1 improves agonist activity at PIEZO1 channels

We replaced the pyrazine ring of Yoda1 with a 2‐, 3‐ or 4‐benzoic acid group (KC157, 2‐benzoic acid; KC158, 3‐benzoic acid; and KC159, 4‐benzoic acid), a 3‐ or 4‐benzamide group (KC162, 3‐benzamide; KC161, 4‐benzamide) or a potassium salt of the 4‐benzoic acid (KC289) (Figure 1a). Each analogue was tested for its ability to evoke intracellular calcium ion (Ca^2+^_i_) elevation in cells overexpressing hPIEZO1, compared directly with Yoda1 at the same concentration of 10 μM (Figure 1b,c). KC159 and KC289 evoke robust responses that are larger than those of Yoda1 (Figure 1b–d). The other analogues fail to evoke responses (Figure 1b,c). Yoda1, KC159 and KC289 fail to evoke responses in a similar cell line that expressed TRPC5 Ca^2+^‐permeable cation channels in place of PIEZO1, suggesting that Ca^2+^ signals need PIEZO1 (Figure S1, SI 1). The selectivity of 5 μM KC289 was further investigated via Eurofins' Hit Profiling Screen PP70 to obtain binding data for 30 proteins including ion channels and receptors (Table S1). There is modest interaction with adenosine A_2A_ and prostanoid EP_4_ receptors and little or no binding to others (Table S1). The data suggest that the 4‐benzoic acid analogue (KC159) and its potassium salt (KC289) are PIEZO1 agonists.

### 3.2 4‐Benzoic acid substitution improves physicochemical properties

Physicochemical properties are important in physiological assays and therapeutics. Kinetic and thermodynamic solubility assays conducted by Malvern Panalytical were used to assess aqueous solubility and other properties. In both, KC159 and KC289 showed better solubility than Yoda1 in phosphate‐buffered saline at physiological pH (Table S2). Our in‐house solubility data suggested that KC159 and KC289 are at least 160 times more soluble in aqueous buffer than Yoda1 (Figure S1, SI 2). A microsomal stability assay performed by Malvern Panalytical yielded half‐lives of 29.6 and 24.6 min, at least 20 times longer than for Yoda1 (Table S2). The fractions of KC159 and KC289 bound to plasma proteins are relatively high (99.4% and 99.35%) but are an improvement on Yoda1 (Table S2). A plasma stability assay indicated good stability over 2 h for Yoda1, KC159 and KC289 (Table S2). The data suggest that key physicochemical properties of KC159 and KC289 are better than those of Yoda1.

### 3.3 4‐Benzoic acid substitution improves concentration–response data for hPIEZO1 channels

**FIGURE 2:**
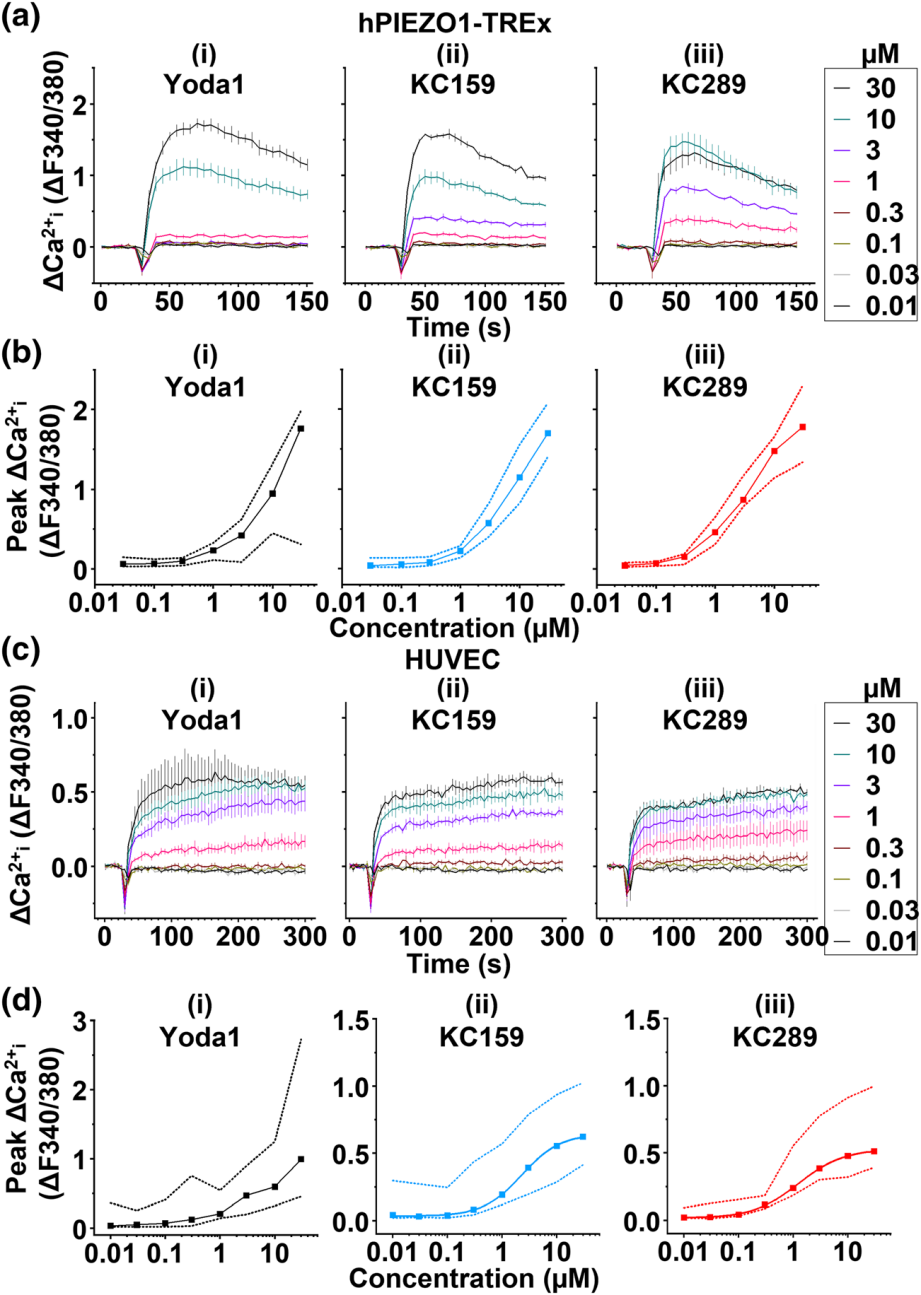
4‐Benzoic acid improves concentration–response data for human PIEZO1. (a) Background‐subtracted (Δ) intracellular Ca^2+^ measurements from a single experiment (n = 1) in which hPIEZO1‐TREx cells were exposed to the indicated concentrations of (i) Yoda1, (ii) KC159 and (iii) KC289. Mean (± SEM) values from three technical replicates are shown. (b) Background‐subtracted (Δ) peak intracellular Ca^2+^ measurements of hPIEZO1‐TREx cells treated with the indicated concentration range of (i) Yoda1, (ii) KC159 and (iii) KC289. Median peak values and the data range from collated experiments are shown (Yoda1, n = 12; KC159, n = 17; and KC289, n = 7). (c) Background‐subtracted (Δ) intracellular Ca^2+^ measurements from a single dose–response experiment (n = 1) in which HUVECs were exposed to the indicated concentrations of (i) Yoda1, (ii) KC159 and (iii) KC289. Mean ± SEM values from three technical replicates are shown. (d) Background‐subtracted (Δ) peak intracellular Ca^2+^ measurements of HUVECs treated with the indicated concentration range of (i) Yoda1, (ii) KC159 and (iii) KC289. Median peak values and the data range from collated experiments are shown (Yoda1, n = 9; KC159, n = 9; and KC289, n = 6). Fitted curves constructed using the Hill equation are shown in (ii) and (iii).

Because of the potential advantages, we investigated KC159 and KC289 in more detail. We first attempted to construct concentration–response curves using cells overexpressing hPIEZO1, comparing the effects of Yoda1, KC159 and KC289 at concentrations up to 30 μM in Ca^2+^ assays. Yoda1 causes concentration‐dependent increases in intracellular Ca^2+^, but a saturating effect is not observed at the highest concentration and its effects are highly variable (Figures 2a,b and S2, SI 1). KC159 and KC289 also do not produce saturating effects, but a saturating inflection point occurs at 10 μM KC289, suggesting that a maximum is approached at approximately 30 μM. The effects of KC159 and KC289 are less variable than those of Yoda1, particularly at high concentrations (Figures 2a,b and S2, SI 1). We performed similar experiments with HUVECs, which are a physiologically relevant cell type that endogenously expresses PIEZO1 channels. Again, the effects of Yoda1 are the most variable, and in this cell system, saturating inflection points occur at 3–10 μM for both KC159 and KC289 (Figures 2c,d and S2, SI 1). Estimated concentrations for 50% effect (EC_50_ values) for KC159 and KC289 are 2.28 and 1.14 μM, respectively (Figure 2d). The data suggest that KC159 and KC289 provide more consistency than Yoda1 in concentration–response studies.

### 3.4 4‐Benzoic acid substitution improves efficacy and potency at mPIEZO1

**FIGURE 3:**
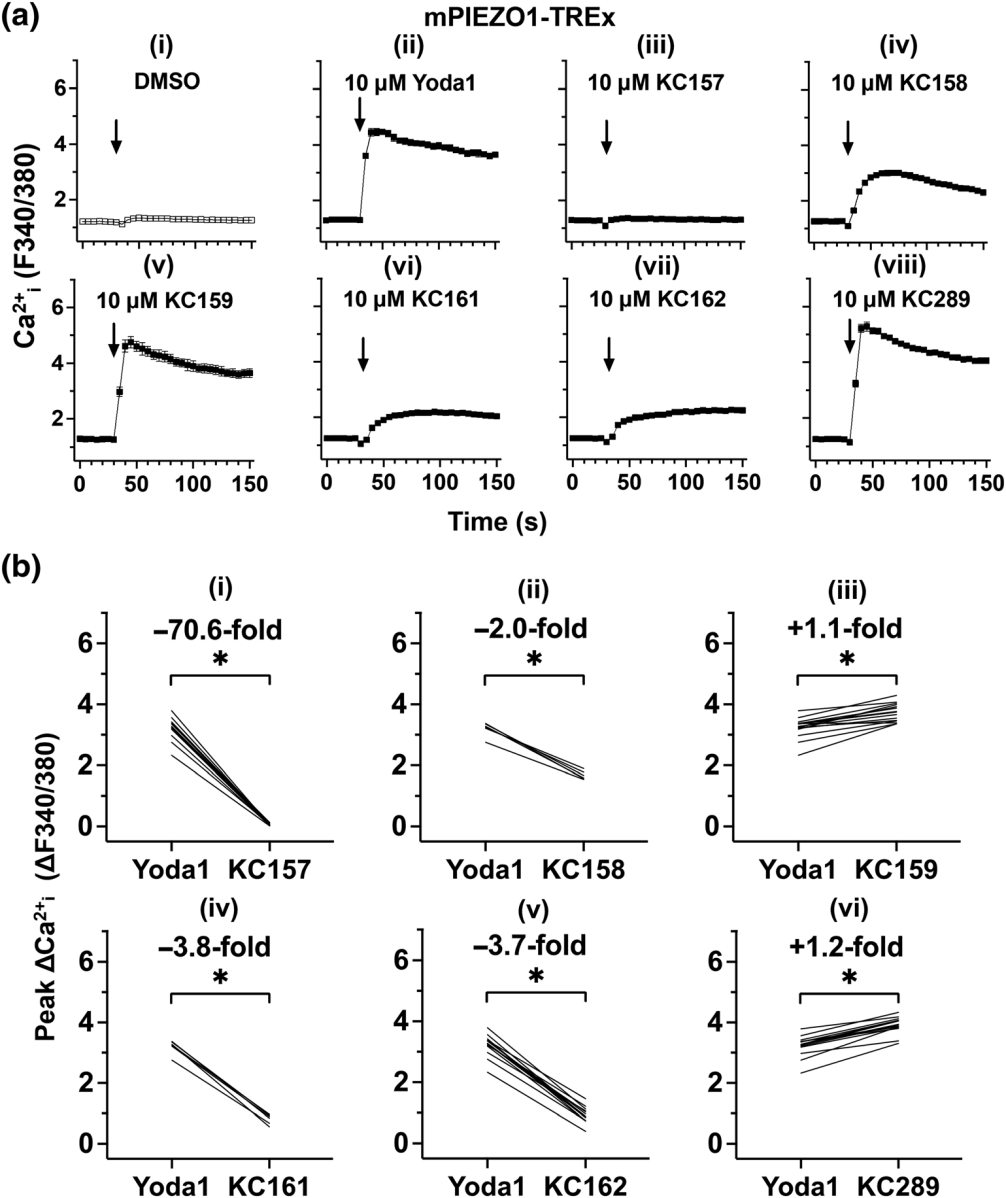
4‐Benzoic acid improves efficacy at mouse PIEZO1. (a) Intracellular Ca^2+^ measurements from a single experiment in which mPIEZO1‐TREx cells were acutely exposed to (i) DMSO vehicle or 10 μM (ii) Yoda1, (iii) KC157, (iv) KC158, (v) KC159, (vi) KC161, (vii) KC162 and (viii) KC289. Arrows indicate the time at which the indicated compound was added to the cells, following a 30 s background read. Mean (± SEM) values from five technical replicates are shown. (b) Paired, background‐subtracted (Δ), peak intracellular Ca^2+^ measurement comparisons of mPIEZO1‐TREx cells treated with 10 μM indicated compound. Each plot shows mean peak values from independent experiments (KC157, n = 15; KC158, n = 6; KC159, n = 15; KC161, n = 6; KC162, n = 15; and KC289, n = 15). Comparisons of the median values of the collated experiments are shown. ^*^ *P* < 0.05, significantly different as indicated; paired‐sample Wilcoxon signed‐rank test. Median fold differences compared to Yoda1 are also indicated.

**FIGURE 4:**
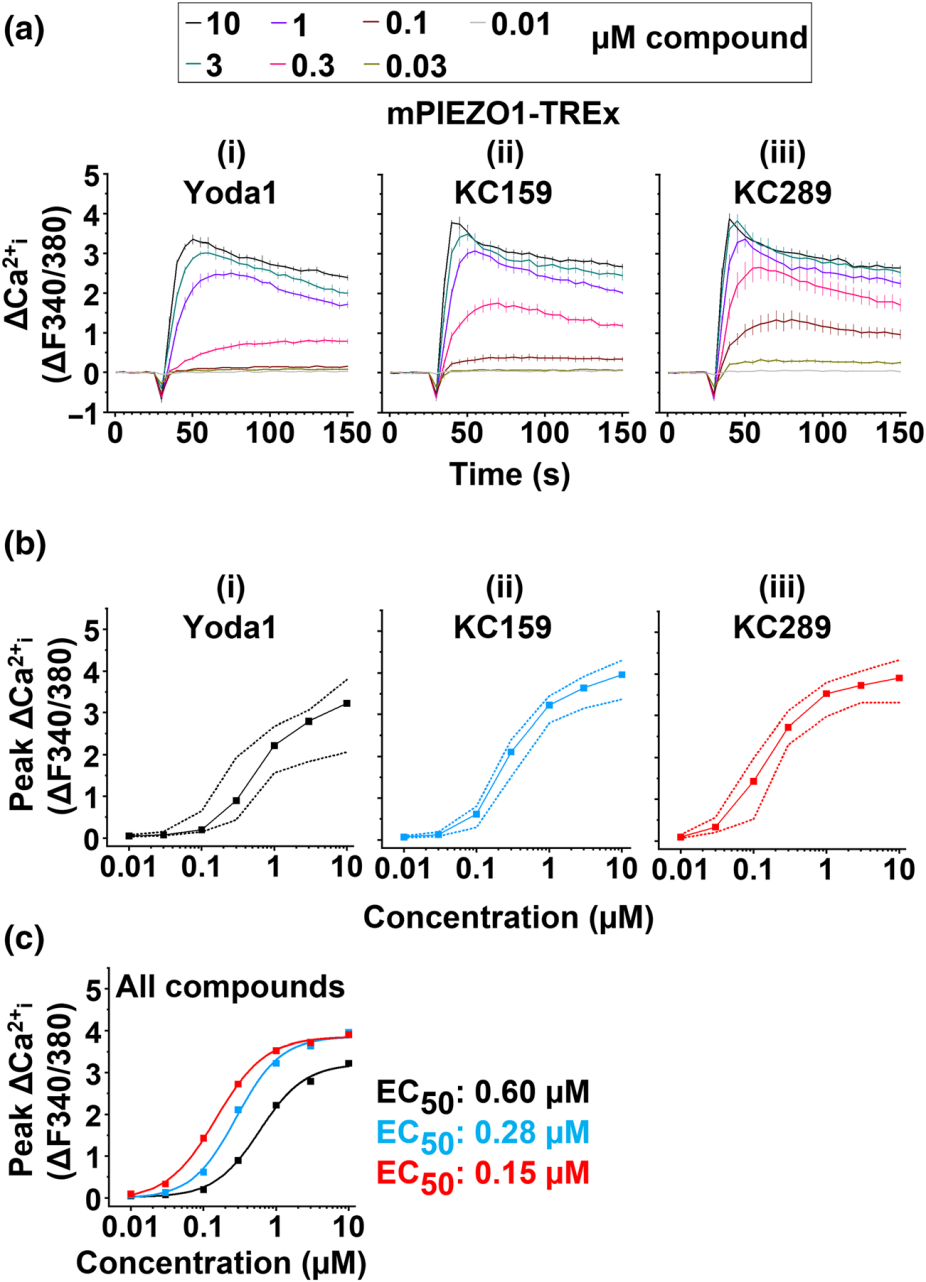
4‐Benzoic acid improves potency at mouse PIEZO1. (a) Intracellular Ca^2+^ measurements from a single experiment (n = 1) in which mPIEZO1‐TREx cells were exposed to the indicated concentrations of (i) Yoda1, (ii) KC159 and (iii) KC289. Mean ± SEM values from four technical replicates are shown. (b) Background‐subtracted (Δ) peak intracellular Ca^2+^ measurements of mPIEZO1‐TREx cells treated with the indicated concentration range of (i) Yoda1, (ii) KC159 and (iii) KC289. Median peak values and the data range from collated experiments are shown (n = 12 for all compounds). (c) Median peak values for each compound were used to construct fitted curves from the Hill equation, and the respective EC_50_ values are indicated.

Previous work suggested that Yoda1 is more potent at mouse, compared with hPIEZO1 channels (Blythe et al., 2019; Evans et al., 2018; Syeda et al., 2015). We therefore studied cells overexpressing mPIEZO1. As with hPIEZO1, KC159 and KC289 evoked larger responses than Yoda1, and KC157 was inactive (Figure 3a,b). KC158, KC161 and KC162 exhibit agonist effects in the mPIEZO1 overexpression system, although their effects are slower to develop and smaller than those of Yoda1 (Figure 3a,b). Concentration–response curves were generated in parallel to directly compare Yoda1, KC159 and KC289. As observed with hPIEZO1 channels, the responses to KC159 and KC289 are less variable than those to Yoda1 (Figures 4a,b and S4, SI 1). In contrast to hPIEZO1, all compounds produce effects that are saturating or approaching saturation at the highest concentrations tested (Figure 4a,b). Therefore, EC_50_s were compared by fitting Hill equations (Figure 4c) and were found to be 0.6 μM for Yoda1, 0.28 μM for KC159 and 0.15 μM for KC289. These data suggest that KC159 and KC289 are more efficacious and potent than Yoda1 at mPIEZO1 channels with a rank order of potency of KC289 > KC159 > Yoda1. KC289 is approximately four times more potent than Yoda1.

### 3.5 Agonist activity is confirmed by manual patch clamp

**FIGURE 5:**
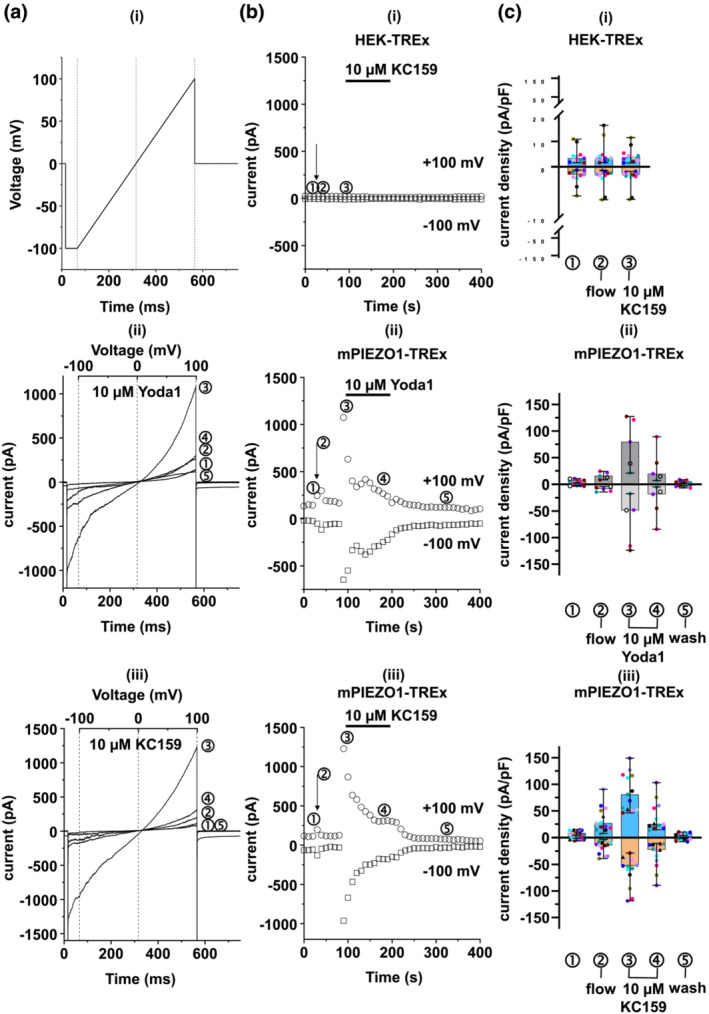
PIEZO1 agonist activity confirmed by manual patch clamp. (a) (i) Ramp voltage protocol used for cell stimulation. Dashed gridlines show time points where the ramp starts (−100 mV), reverses (0 mV) and ends (+100 mV). They also match the lines in (ii) and (iii), which show representative (n = 1) whole‐cell currents activated in mPIEZO1‐TREx channels by 10 μM (ii) Yoda1 or (iii) KC159. The circled numbers indicate currents at the following stages of the experiment: ① control, ② after the start of SBS flow, ③ (maximal) after application of the indicated compound, ④ 100 ms after application of compound and ⑤ after bath solution (SBS) washout of compound. The circled numbers correspond to those shown in all subsequent plots. (b) Time‐resolved plots of representative experiments (n = 1): (i) HEK‐TREx control (null) cell treated with fluid flow and then flow plus 10 μM KC159; (ii) mPIEZO1‐TREx cell treated with flow and then flow plus 10 μM Yoda1; and (iii) mPIEZO1‐TREx cell treated with flow and then flow plus 10 μM KC159. Arrow indicates the start of SBS flow. Circles indicate currents measured at +100 mV of the ramp, and squares indicate those measured at −100 mV. The currents were recorded every 10 s. (c) Collated data for experiments as described in (a) and (b). Current densities (pA·pF^−1^) measured at +100 mV are shown on the upper Y axis (blue for KC159 and dark grey for Yoda1) and those measured at −100 mV on the lower Y axis (orange for KC159 and light grey for Yoda1). Individual data points of different cells are shown with different colours. Bars indicate the median ± range. (i) HEK‐TREx control cells treated with 10 μM KC159: ① n = 11, ② n = 11 and ③ n = 11. (ii) mPIEZO1‐TREx cells treated with 10 μM Yoda1: ① n = 5, ② n = 5, ③ n = 5, ④ n = 5 and ⑤ n = 5. (iii) mPIEZO1‐TREx cells treated with 10 μM KC159: ① n = 13, ② n = 13, ③ n = 13, ④ n = 13 and ⑤ n = 6

An important technique in ion channel studies is manual patch clamp. Its throughput is limited, and so we focused on a comparison of Yoda1 and KC159 in cells overexpressing mPIEZO1. Mechanical stimulus due to fluid flow through the recording chamber causes a small current in PIEZO1‐expressing but not in control (i.e., null) cells that lacked transfection with PIEZO1 (Figure 5a–c, i cf. ii and iii). KC159 increases current in PIEZO1‐expressing but not null cells, seen as outward current at positive voltages and inward current at negative voltages, with reversal near 0 mV as expected for PIEZO1 currents (Figure 5b,c, i and iii). The effect is similar to that of Yoda1 (Figure 5b,c, ii). Current reaches a peak and then declines to a plateau in the continuous presence of KC159 or Yoda1 (Figure 5b,c, ii and iii). The plateau current disappears when KC159 or Yoda1 is washed from the chamber (Figure 5b,c, ii and iii). The application of DMSO only (the solvent for Yoda1 and KC159) evoked no change in current in six independent recordings from cells overexpressing mPIEZO1. The data further suggest that KC159 is an agonist of PIEZO1. They suggest that the responses to a single high concentration are similar to those of Yoda1.

### 3.6 Improved agonist activity is confirmed by high‐throughput patch clamp

**FIGURE 6:**
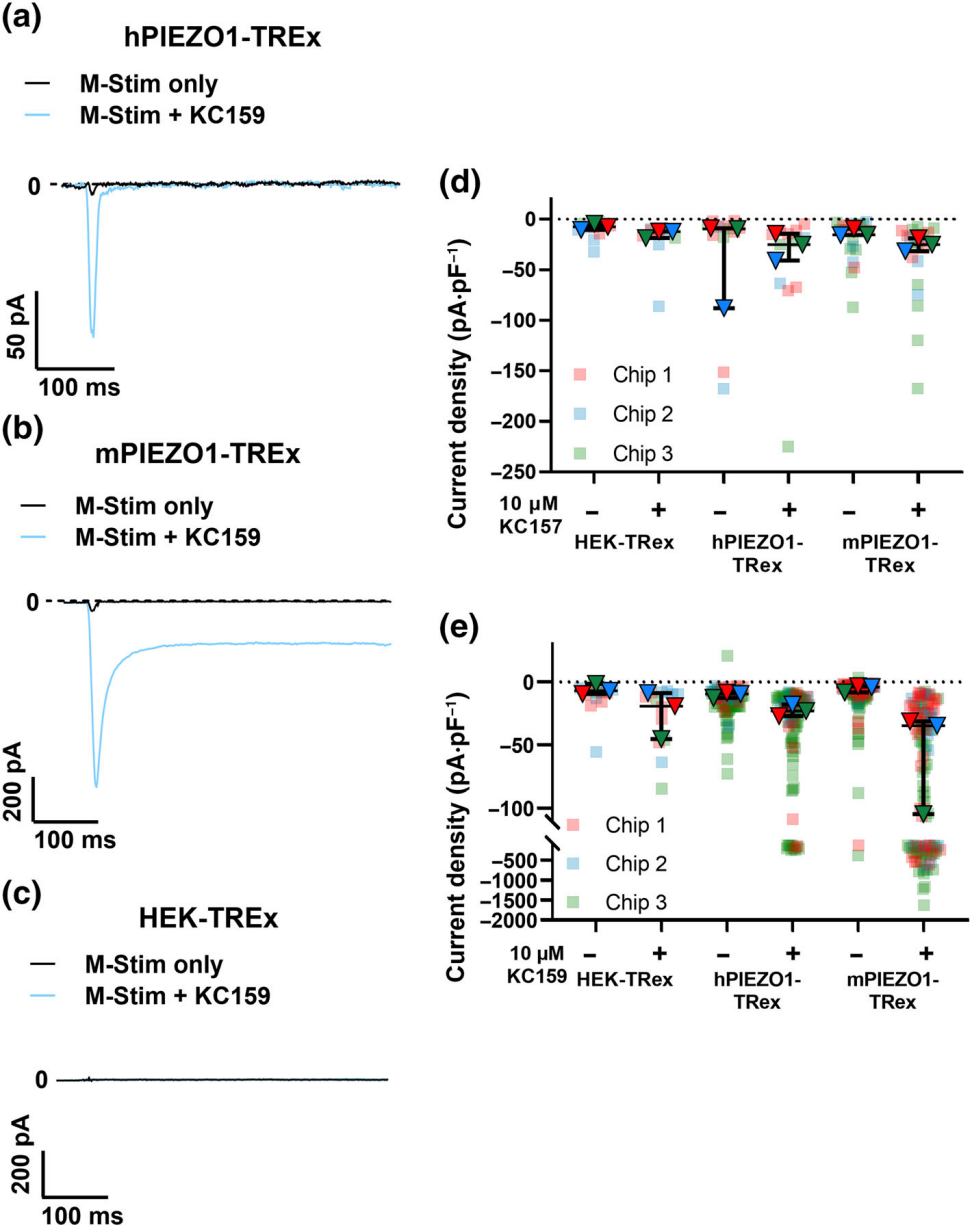
Improved agonist activity as measured by automated patch clamp. Averaged traces for PIEZO1‐mediated currents activated by mechanical stimulation (M‐Stim) on the SyncroPatch 384 in the absence (black traces) and presence of 10 μM KC159 (blue traces) from (a) hPIEZO1‐TREx (n = 44), (b) mPIEZO1‐TREx (n = 46) and (c) untransfected HEK‐TREx (n = 55). Current densities elicited by M‐Stim in the absence and presence of 10 μM (d) KC157 or (e) KC159 in untransfected HEK‐TREx, hPIEZO1‐TREx and mPIEZO1‐TREx cells. Each square represents the peak current density of a single cell, with median values and ranges indicated in black. Only data for cells defined as responding (see Section 2) are included in the plots. Recordings were made from cells on three different 384‐well recording chips, and the data from these chips are distinguished by red, blue and green colours with the filled triangles indicating the median values for each chip. (d) HEK‐TREx M‐Stim (n = 10), HEK‐TREx M‐Stim + KC157 (n = 10), hPIEZO1‐TREx M‐Stim (n = 12), hPIEZO1‐TREx M‐Stim + KC157 (n = 12), mPIEZO1‐TREx M‐Stim (n = 20) and mPIEZO1‐TREx M‐Stim + KC157 (n = 20). (e) HEK‐TREx M‐Stim (n = 13), HEK‐TREx M‐Stim + KC159 (n = 13), hPIEZO1‐TREx M‐Stim (n = 94), hPIEZO1‐TREx M‐Stim + KC159 (n = 94), mPIEZO1‐TREx M‐Stim (n = 113) and mPIEZO1‐TREx M‐Stim + KC159 (n = 113)

For comparative electrophysiological studies, we performed high‐throughput automated patch clamp on cells overexpressing hPIEZO1 or mPIEZO1, relative to null cells that lacked the overexpression of PIEZO1. Mechanical stimulation alone (M‐Stim only) was caused by fluid flow from the compound application pipette, which we expected would cause shear stress on the cells and possibly also a compression force. M‐Stim only elicits small transient inward currents at the holding potential of −80 mV in cells expressing human or mPIEZO1 (Figure 6a–e). The addition of 10 μM KC159 greatly amplifies the currents (Figure 6a–e). In mPIEZO1 cells, the currents show a sustained component that is 26 ± 8% (mean ± SD) of the peak current amplitude, which is not seen in hPIEZO1 cells (−4 ± 2% of peak current) (Figures 6b and S6, SI 1). There are little or no responses in empty cells (Figure 6c–e). KC157 mostly fails to evoke responses (Figures 6d and S6, SI 1).

**FIGURE 7:**
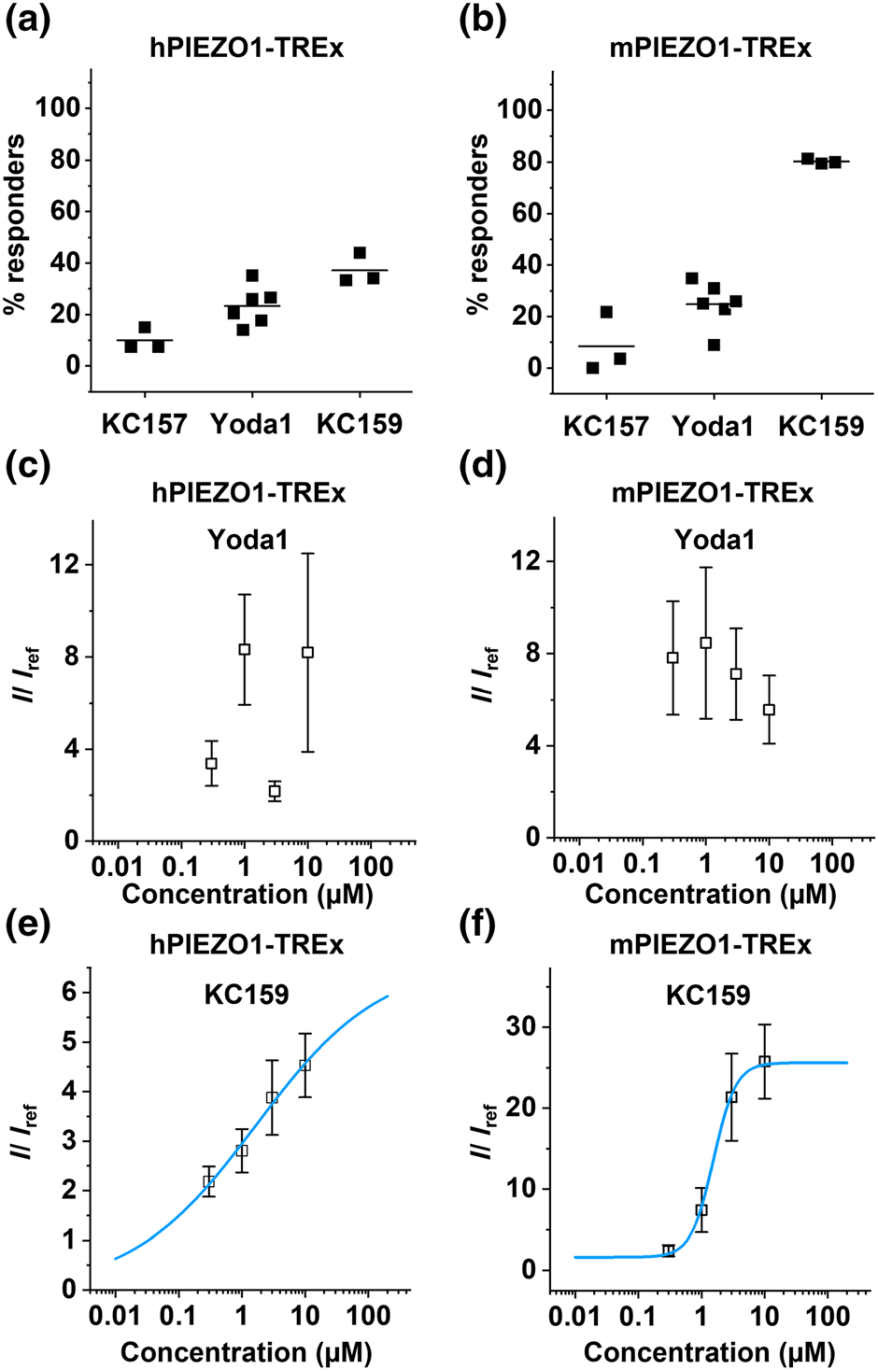
Automated patch‐clamp comparison with Yoda1. Percentages of (a) hPIEZO1‐TREx and (b) mPIEZO1‐TREx cells responding to activation by 10 μM KC157, 10 μM Yoda1 and 5 μM KC159. Each square represents an independent experiment with mean values indicated by black bars. All groups contained 64 cells per condition tested on the same chip, and the following numbers of valid cells were assessed for responsiveness in each repeat: (a) KC157, n = 40, 40 and 53; Yoda1, n = 37, 50, 51, 46, 39 and 49; and KC159, n = 42, 50 and 41. (b) KC157, n = 48, 55 and 46; Yoda1, n = 42, 48, 56, 48, 43 and 54; and KC159, n = 34, 25 and 43. Because the number of independent 384‐well chips used was less than five in some cases (i.e., three), we did not apply statistical testing to these data. Dose–response data expressed as mean normalised activation ± SEM, obtained from (c) hPIEZO1‐TREx (n = 108) and (d) mPIEZO1‐TREx (n = 134) cells exposed to increasing concentrations (0.3, 1, 3 and 10 μM) of Yoda1. hPIEZO1 0.3 μM (n = 27), 1 μM (n = 25), 3 μM (n = 28) and 10 μM (n = 28); mPIEZO1 0.3 μM (n = 34), 1 μM (n = 34), 3 μM (n = 34) and 10 μM (n = 32). The peak current in the presence of the agonist was normalised to the peak current in the presence of reference only. Dose–response data expressed as mean normalised activation ± SEM, obtained from (e) hPIEZO1‐TREx (n = 316) and (f) mPIEZO1‐TREx (n = 250) cells exposed to increasing concentrations (0.3, 1, 3 and 10 μM) of KC159. hPIEZO1 0.3 μM (n = 79), 1 μM (n = 86), 3 μM (n = 73) and 10 μM (n = 78); mPIEZO1 0.3 μM (n = 65), 1 μM (n = 58), 3 μM (n = 63) and 10 μM (n = 64). The peak current in the presence of the agonist was normalised to the peak current in the presence of reference only. Fitted curves (blue) were generated from the Hill equation.

We next performed paired comparisons with Yoda1. KC159 causes more cells to respond compared with Yoda1, most notably for mPIEZO1 (Figure 7a,b). KC157 is largely ineffective (Figure 7a,b). Yoda1 responses are highly variable, and concentration dependence is lacking (Figure 7c,d). KC159 elicits concentration‐dependent increases in current in hPIEZO1 and mPIEZO1 cells (Figure 7e,f). Maximum responses are not certain, and so EC_50_s are only estimates at 1.67 and 1.54 μM for hPIEZO1 and mouse PIEZO, respectively. The data suggest that KC159 is more reliable and effective as a PIEZO1 channel agonist than Yoda1, and more effective at mPIEZO1 channels that at hPIEZO1 channels.

### 3.7 KC159 and KC289 relax the mouse portal vein

**FIGURE 8:**
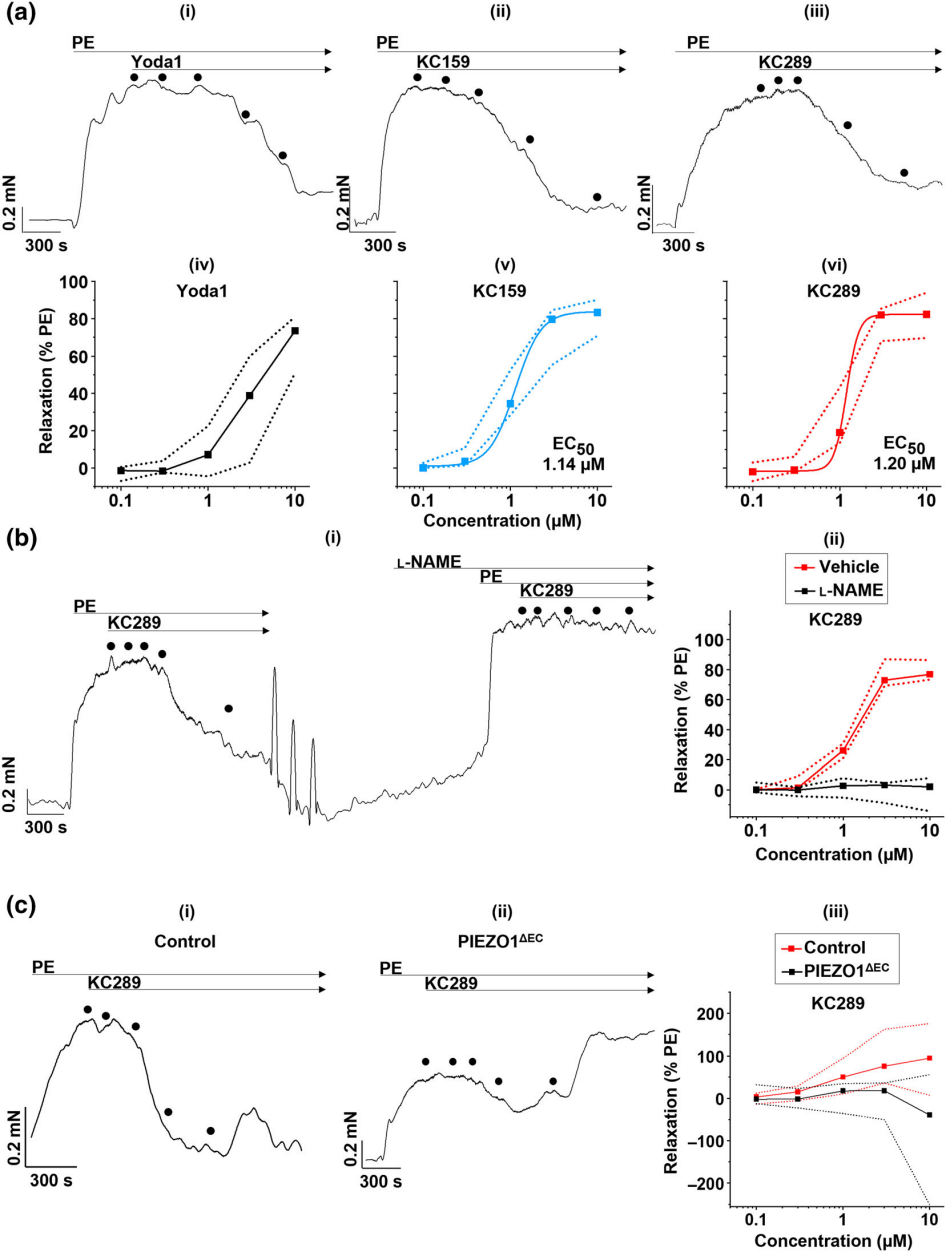
NO‐ and endothelial PIEZO1‐dependent vasorelaxation. Isometric tension of mouse portal vein possessing intact endothelium was measured following pre‐constriction with 10 μM phenylephrine (PE) and exposure to increasing concentrations (0.1, 0.3, 1, 3 and 10 μM) of indicated compounds. (a) (upper panel) Isometric tension traces from single experiments (n = 1) in which mouse portal vein segments were exposed to increasing concentrations of (i) Yoda1, (ii) KC159 and (iii) KC289. The dots indicate the sequential addition of rising concentrations of compounds. (lower panel) Corresponding dose–response data for (iv) Yoda1 (n = 8), (v) KC159 (n = 5) and (vi) KC289 (n = 5) expressed as a % of the maximal PE‐induced tension. Squares indicate median values, and dotted lines indicate the data range from collated experiments. For Yoda1, curve fitting was not successful due to the lack of upper plateau. For KC159 and KC289, fitted curves generated from the Hill equation (Hill1 in OriginPro 2020 software) and their corresponding EC_50_ values are shown. (b) (i) Isometric tension response trace from a single experiment (n = 1) in which increasing concentrations of KC289 were added before and after a 30 min pre‐incubation of the vessel with 100 μM l‐NAME. (ii) Corresponding collated dose–response data expressed as a % of the maximal PE‐induced tension (n = 5). Median and range are shown. (c) Isometric tension response traces from a single experiment (n = 1) in which increasing concentrations of KC289 were added to portal veins from (i) control and (ii) PIEZO1^ΔEC^ mice. (iii) Corresponding dose–response data expressed as a % of the maximal PE‐induced tension (control, n = 9; PIEZO1^ΔEC^, n = 9). Median and range from collated experiments are shown.

Previous studies have suggested that PIEZO1 channels signal to endothelial NO synthase (NOS3) to generate NO and thereby cause endothelium‐dependent relaxation in blood vessels (Beech & Kalli, 2019). We used this effect to investigate KC159 and KC289 in a physiological assay. The portal vein was studied because of evidence that PIEZO1 channels are important in the hepatic circulation (Caolo et al., 2020; Hilscher et al., 2019; Li et al., 2014; Rode et al., 2017). Vessel wall tension was recorded ex vivo using wire myography, and smooth muscle contraction was evoked by the α_1_‐adrenoceptor agonist phenylephrine (PE) so that endothelium‐dependent relaxation could be observed (e.g., Figure 8a). Yoda1, KC159 and KC289 caused concentration‐dependent relaxation (Figure 8a). KC159 and KC289 concentration–response curves reach saturation, thus enabling calculation of EC_50_s of 1.14 and 1.20 μM, respectively (Figure 8a). Yoda1 effects are more variable, and no saturation occurs (Figure 8a). The data suggest that KC159 and KC289 are suitable for use in a physiological assay and are an improvement on Yoda1.

### 3.8 KC289‐evoked relaxation is NO dependent

To investigate if KC289 causes relaxation via NOS3, we incubated portal vein with l‐NAME, which is a substrate inhibitor of NOS3. l‐NAME abolishes the relaxant effect of KC289 (Figure 8b). Effects of l‐NAME in the absence of KC289 are shown in Figure S8 (SI 1). We had technical difficulty removing endothelium from portal vein without damaging the smooth muscle layer, and so we could not test the endothelium dependence of the KC289 response. The data suggest that KC289 acts to stimulate NOS3 and NO production, as expected for an agonist at PIEZO1 channels.

### 3.9 KC289‐evoked relaxation is dependent on endothelial PIEZO1 channels

To determine the role of endothelial PIEZO1 channels in KC289 responses, we compared samples of portal vein taken from control mice with those from matched mice in which endothelial PIEZO1 expression was conditionally deleted at the adult stage (PIEZO1^ΔEC^), as previously described (Caolo et al., 2020; Rode et al., 2017). KC289‐evoked relaxation is inhibited by endothelial PIEZO1 deletion, and a contractile response often becomes evident at 10 μM (Figure 8c). Variable spontaneous oscillatory contractions occurred in these experiments, regardless of PIEZO1 deletion. This may explain the wide variability in the overall data and response to KC289. For transparency, all original traces are provided (Figure S8, SI 2). The mechanism of the contractile effect of KC289 was not investigated, but it may relate to PIEZO1 expressed in smooth muscle cells or another cell type in the vessel.

### 3.10 KC289‐evoked relaxation is inhibited by Dooku1

Dooku1 is an analogue of Yoda1 that antagonises the action of Yoda1 (Evans et al., 2018). Pre‐incubation with 10 μM Dooku1 reduces relaxations evoked by KC159 and KC289 (Figure S8, SI 3). Dooku1 also consistently inhibited responses to phenylephrine (Figure S8, SI 3), as previously reported in studies of mouse aorta (Evans et al., 2018). The data suggest that KC159 and KC289 cause relaxation via a site that is the same as that mediating the effects of Yoda1.

### 3.11 Effects of 3‐ and 4‐benzamide and 3‐benzoic acid analogues

**FIGURE 9:**
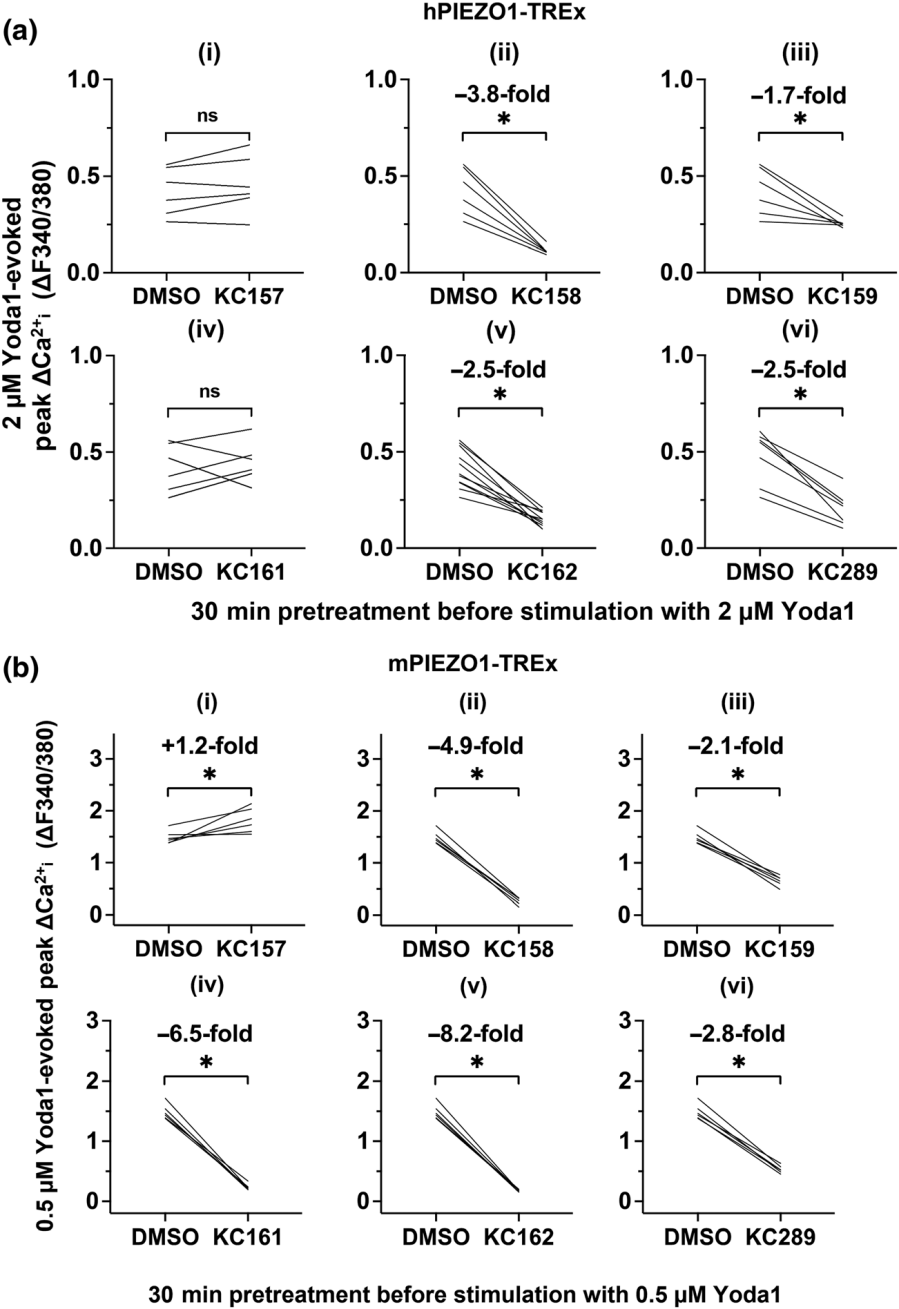
Effects of 3‐ and 4‐benzamide and 3‐benzoic acid analogues. Paired, background‐subtracted (Δ), peak intracellular Ca^2+^ measurement comparisons of (a) hPIEZO1‐TREx and (b) mPIEZO1‐TREx pretreated with 10 μM indicated compound for 30 min prior to acute exposure to (ii–vi) the indicated Yoda1 stimulus. Each plot shows mean peak values from independent experiments. (a) Independent experiment numbers for hPIEZO1‐TREx experiments: KC157, n = 6; KC158, n = 6; KC159, n = 6; KC161, n = 6; KC162, n = 11; and KC289, n = 7. (b) Independent experiment numbers for mPIEZO1‐TREx experiments: n = 6 for all compounds. Comparisons of the median values of the collated experiments are shown. ^*^ *P* < 0.05, significantly different as indicated; ns, not significantly different; paired‐sample Wilcoxon signed‐rank test. Median fold differences compared to DMSO pretreatment/indicated Yoda1 stimulation are also shown.

In view of the effect of Dooku1 described above, we tested if there are inhibitory actions of the new Yoda1 analogues by pre‐treating cells at 10 μM for 30 min prior to adding 2 μM Yoda1 (Figures 9 and S9, SI 1). All except KC157 and KC161 inhibited the action of Yoda1 on hPIEZO1 channels (Figures 9a and S9, SI 1). Only KC157 failed to inhibit the action of Yoda1 on mPIEZO1 channels (Figures 9b and S9, SI 1). KC159 and KC289 were agonists, and so they may inhibit the effect of Yoda1 simply by pre‐activating the channels. KC158, KC161 and KC162 are also agonists of mPIEZO1 channels (Figure 3). KC158 and KC162 appear to inhibit the Yoda1 response without pre‐activating hPIEZO1 (Figures 9a, S9, SI 1 and 1b,c), but we noticed an elevated baseline Ca^2+^ signal for KC161 and KC162 in the pre‐incubation protocol, suggesting that these compounds are actually slowly acting mild agonists (Figure S9, SI 2). The data suggest that 3‐benzamide (KC162), 4‐benzamide (KC161) and 3‐benzoic acid (KC158) analogues are mild, slowly acting, agonists that may also inhibit the effect of Yoda1, as if they were partial agonists. Only the 2‐benzoic acid analogue (KC157) lacks agonist or antagonist properties.

### 3.12 Relationship to PIEZO2

**FIGURE 10:**
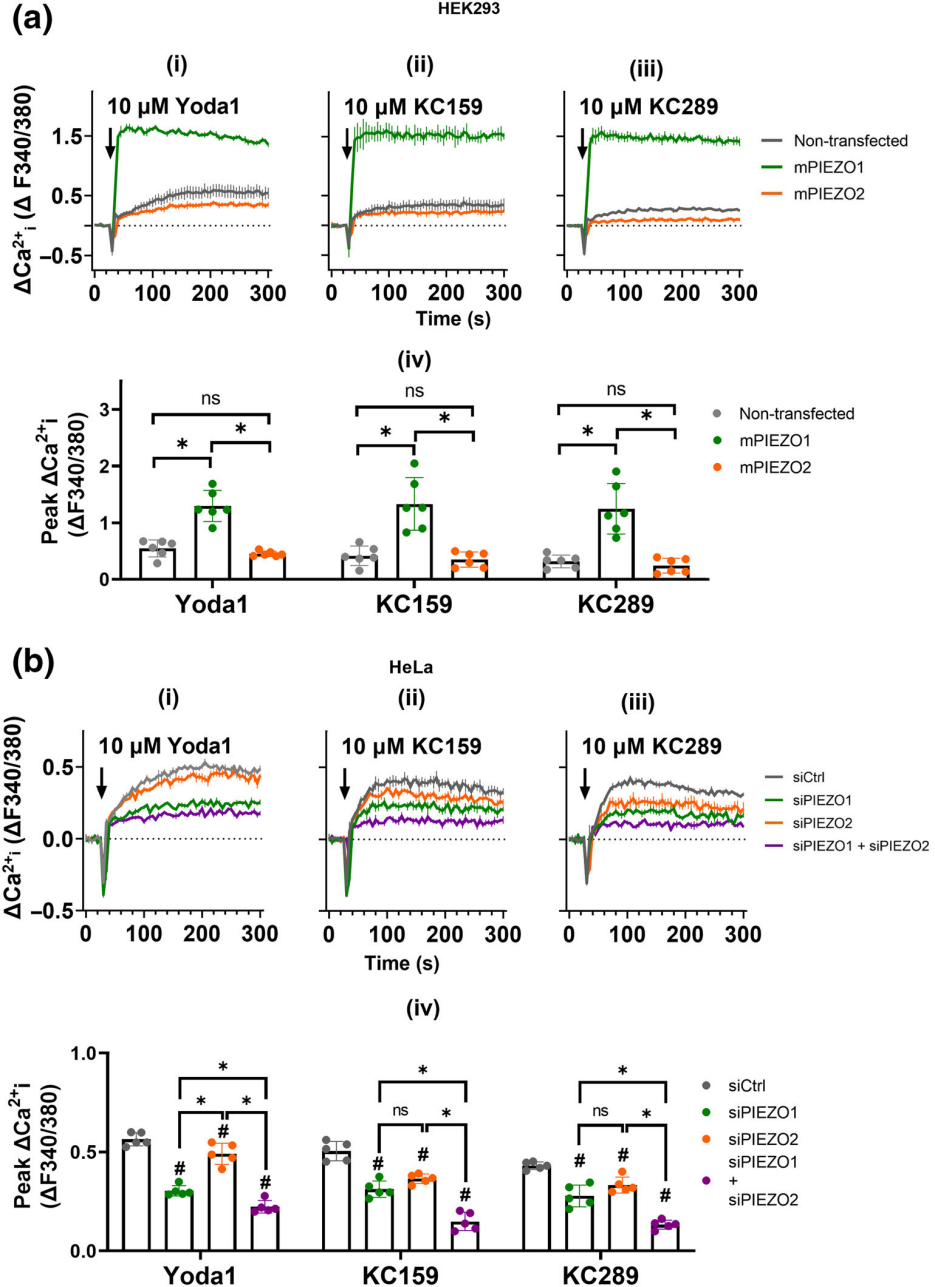
Relevance to PIEZO2. (a) Background‐subtracted (Δ) peak intracellular Ca^2+^ measurements of HEK 293 cells overexpressing mPIEZO1, mPIEZO2 or non‐transfected cells. Cells were exposed to 10 μM (i) Yoda1, (ii) KC159 and (iii) KC289. In each case, mean ± SEM values from four technical replicates are shown (n = 1 each). (iv) Mean ± SD (n = 6) for peak Ca^2+^ signals evoked by 10 μM Yoda1, KC159 and KC289. (b) Background‐subtracted (Δ) peak intracellular Ca^2+^ measurements of HeLa cells after siRNA mediated knockdown of hPIEZO1 (siRNA targeted to PIEZO1 expression [siPIEZO1]), hPIEZO2 (siPIEZO2), both (siPIEZO1 + siPIEZO2) or transfected with a non‐targeting (control [Ctrl]) siRNA (siCtrl). Cells were exposed to 10 μM (i) Yoda1, (ii) KC159 and (iii) KC289. Mean ± SEM values from four technical replicates are shown (n = 1 each). (iv) Mean ± SD (n = 5) for peak Ca^2+^ signals evoked by 10 μM Yoda1, KC159 and KC289. Mean values of the collated experiments are shown. ^*^ *P* < 0.05, significantly different as indicated; ns, not significantly different; ^#^ *P* < 0.05, significantly different from siCtrl; repeated measures one‐way ANOVA followed by Tukey's post hoc test for multiple comparisons.

Yoda1 was discovered in a chemical screen of HEK 293 cells overexpressing mPIEZO1 and mPIEZO2, but Yoda1 was subsequently found not to activate mPIEZO2 overexpressed alone (Syeda et al., 2015). To investigate KC159 and KC289 in relation to PIEZO2, we compared HEK 293 cells overexpressing mPIEZO1, mPIEZO2 or neither (‘non‐transfected cells’) (Figures 10 and S10, SI 1 and 2). Ca^2+^ elevations evoked by KC159 or KC289 in HEK 293 cells overexpressing mPIEZO2 are not different from background signals in non‐transfected cells and contrast with the larger responses when mPIEZO1 was overexpressed (Figure 10a). The background signals may have been due to endogenous hPIEZO1 channels of these HEK 293 cells (Dubin et al., 2017). To investigate the relevance to native PIEZO2 channels, we studied HeLa cells because they natively express PIEZO2 (The Human Protein Atlas, 2022). Yoda1, KC159 and KC289 evoked Ca^2+^ elevations in HeLa cells (Figure 10b). To determine the relevance to PIEZO2, we used RNA interference to deplete its expression (Figure S10, SI 2). There is PIEZO1 in HeLa cells (Geng et al., 2020), and so we depleted its expression also (Figure S10, SI 2). PIEZO2 depletion partly suppressed responses to KC159, KC289 and Yoda1, but with greater effect on responses to KC159 and KC289 (Figure 10b). Responses to Yoda1, KC159 and KC289 are all partly suppressed by PIEZO1 depletion (Figure 10b). Depletion of PIEZO1 and PIEZO2 further suppresses the responses (Figure 10b). The data suggest that, like Yoda1, KC159 and KC289 are not agonists of overexpressed mPIEZO2 but may act as agonists at native human PIEZO2 channels, at least when they are natively co‐expressed with PIEZO1 channels.

## 4 DISCUSSION

In this study, we modified the chemical structure of Yoda1 with the aim of increasing the ability to modulate PIEZO1 channels, in physiological settings. Our data suggest that changes to the pyrazine moiety in Yoda1 is a route to improving both the agonist activities and the physicochemical properties. Specifically, benzoic acid substitution with carboxylate is beneficial, with the 4‐position being critical. Substitution at the 2‐position generates an inactive compound whereas the 3‐position leads to inferior agonism. The 2‐position analogue can serve as a negative control comparator compound. When there is 4‐benzamide instead of 4‐benzoic acid, there is weak or no agonist activity. Because 4‐benzoic acid and 4‐benzamide occupy a similar molecular volume, it may be the neutral charge of 4‐benzamide at physiological pH that impairs agonist activity. The potassium salt of the 4‐benzoic acid analogue (KC289) is more effective than the analogue itself (KC159). Its improved aqueous solubility is likely to contribute to this improvement. Nevertheless both KC159 and KC289 are advances on Yoda1 in terms of agonist activity at PIEZO1 channels.

An important next step would be the determination of the binding site(s) for Yoda1, KC159 and KC289, particularly if such a site or sites could be characterised at atomic resolution. There could then be better rational design and understanding of PIEZO1 modulators. Surface plasmon resonance studies suggest binding of Yoda1 between residues 1 and 2190 of mPIEZO1 (in total, it comprises 2547 residues) (Wang et al., 2018). Computer simulations have suggested a pocket away from the C‐terminal region of the central ion pore and mutagenesis data point to importance of residues such as alanine at position 1718 (Botello‐Smith et al., 2019). KC289 could help future structural determination studies by enabling incubation of PIEZO1 with a higher concentration of compound and perhaps conferring stronger binding. Our data suggest a greater effect and potency of KC159 and KC289 at mPIEZO1, compared with hPIEZO1, so mPIEZO1 may be the most promising route to binding site determination. Moreover, comparisons of mouse and human sequences could indicate what is optimal for binding and efficacy. However, we suggest caution in such inferences because the expression of mPIEZO1 may have been better than hPIEZO1 in our studies, possibly creating an impression of greater efficacy and sensitivity through greater receptor reserve.

A variety of factors could explain differences in apparent efficacy and potency. In addition to receptor reserve, aqueous solubility could be important. Our estimates suggest aqueous solubility of Yoda1 only up to the low μM range. We used it up to 30 μM, and other groups report use of higher concentrations. Inclusion of a solvent such as DMSO in the buffer (as we did) may aid solubility, but partial precipitation could still occur and potentially explain variability in Yoda1 effects. KC159 and KC289 have better aqueous solubility, and so this may be why they show less variability in effect. Another factor contributing to differences in apparent efficacy and potency could be the complexity of the transduction pathway between channel activation and effect. When tested against overexpressed mPIEZO1 in Ca^2+^ assays, the EC_50_s for KC289 and KC159 were 0.15 and 0.28 μM, whereas in mouse portal vein relaxation, the EC_50_s were 1.2 and 1.14 μM. The portal vein effects occur via PIEZO1, and so a high concentration of compound may be required to overcome non‐linearity in the signalling.

The EC_50_ we report for Yoda1 is lower (i.e., ‘better’) than that reported in some other studies (Lacroix et al., 2018; Syeda et al., 2015). There could be several reasons, including different channel expression levels and experimental conditions, but we note that we found considerable variability in the effect of Yoda1, such that we also sometimes observed high EC_50_s in individual experiments. We suggest that such differences may be due to the limited aqueous solubility of Yoda1 at concentrations relevant to EC_50_ determination, causing Yoda1 to variably precipitate out of solution. In support of this hypothesis, our new, more soluble, analogues had less variable effects.

Manual patch clamp enables comparisons of the efficacy and potency of compounds, but its utility is restricted by the low throughput of recording from one cell at a time. Therefore, we explored automated patch‐clamp technology, which has the potential to increase throughput greatly. The derived data support our findings from 96‐well Ca^2+^ measurements by suggesting that KC159 has advantages over Yoda1 and that KC157 (the 2‐position analogue) is inactive. Differences between mPIEZO1 and hPIEZO1 are again evident. In this regard, we observed an intriguing sustained response of mPIEZO1 but not hPIEZO1. In these experiments, there was a rapid small‐volume application of compound followed by rapid retraction of double the volume. Reversal of the KC159 effect occurs after washout in manual patch recordings, but these recordings were on a slower timescale. It is possible that the rapid retraction in the automated system only partly removes KC159 in the short time frame of the recordings, leaving low concentrations of compound. This low concentration may cause the sustained current evident in mPIEZO1 but not hPIEZO1 because KC159 showed greater efficacy and potency in experiments with mPIEZO1. This hypothesis is supported by failure to observe sustained current when applying Yoda1, which is less effective than KC159.

A limitation of our efficacy comparison in Figure 3 is that the effects of the various analogues may not have been compared at equal saturation for all compounds. This is challenging to overcome because of the limited aqueous solubility of this series of compounds, despite our improvements. Therefore, we cannot exclude the possibility that some of the compounds would be more effective if tested at higher concentrations, if they were to be retained in aqueous solution.

Yoda1, KC159 and KC289 are tool compounds for laboratory and potentially in vivo animal studies aimed at determining functions of PIEZO1 and informing potential drug discovery projects. We do not yet know if they have therapeutic relevance or suitability (e.g., distribution and safety features) for use in humans. Nevertheless, there is potential clinical value. A disease area to consider is malaria because gain‐of‐function mutation in PIEZO1 is associated with protection against malaria (Ma et al., 2018), and so this effect might be mimicked by a PIEZO1 agonist acting on wild‐type PIEZO1. Another area to consider is lymphoedema because loss‐of‐function mutations in PIEZO1 are associated with generalised lymphatic dysplasia (Fotiou et al., 2015), and so PIEZO1 agonists might be beneficial if partly functional channels are still available.

Despite Yoda1's value and apparent selectivity, its use at relatively high concentrations could make it especially vulnerable to as‐yet unknown off‐target effects. Moreover, KC159 and KC289 are analogues of Yoda1, but they are not the same chemicals and may not have the same selectivity profile. We provide evidence for their selectivity: There is little or no activation of Ca^2+^ or electrophysiological signals in control (null) HEK 293 cells without PIEZO1 overexpression; they do not activate mPIEZO2 or human TRPC5 overexpressed in HEK 293 cells; genetic disruption of PIEZO1 inhibits vasorelaxation; and a commercial selectivity screen suggests little or no binding to a range of other proteins other than potential modest effects on A_2A_ and EP_4_ receptors (Table S1). We did not further investigate the relevance of the latter receptors, but the strong inhibitory effect of genetic disruption of PIEZO1 on the effect of KC289 suggests that PIEZO1‐independent effects do not contribute, or are, at most, minor contributors in portal vein.

Although KC159 and KC289 did not activate overexpressed mPIEZO2 channels, knockdown of native human PIEZO2 in HeLa cells partly inhibited responses to KC159 and KC289. These data suggest that the new Yoda1 analogues (such as KC289) may have the ability to activate PIEZO2 channels in a species‐ or context‐dependent manner. This warrants further investigation using electrophysiological approaches and overexpressed human PIEZO2 because there are currently limited possibilities for chemically modulating PIEZO2 channels. Although siPIEZO2 had no significant effect on PIEZO1 mRNA abundance, there was a visual impression of a potential small off‐target effect on PIEZO1 (Figure S10, SI 1B). This does not detract from the observation that siPIEZO2 is more effective against responses evoked by KC159 and KC289 than Yoda1 (Figure 10b). Therefore, we cautiously suggest that some Yoda1 analogues may cross over to PIEZO2 and thereby provide a route to PIEZO2 agonists.

In conclusion, this study supports the idea that Yoda1 provides a template for developing a series of useful PIEZO1 channel modulators. The structure–activity requirements are quite strict and seem to be relatively inflexible at the 2,6‐dichlorophenyl moiety (Evans et al., 2018), but our data suggest better opportunities at the pyrazine moiety. Here, we show the value of 4‐benzoic acid substitution and improvement on Yoda1 for PIEZO1 agonist activity in terms of efficacy, potency and physicochemical properties (such as aqueous solubility). We suggest calling the potassium salt of this analogue (KC289) Yoda2 and propose its potential value as a tool compound in physiological assays and for facilitating efforts to identify a binding site. In addition, we suggest consideration of Yoda2 or a variant thereof in disease conditions such as malaria and lymphoedema.

## CONFLICTS OF INTEREST

Automated patch‐clamp studies were performed at Nanion Technologies GmbH, which has interest in the commercial success of the SyncroPatch 384. Authors at Leeds and Homburg have interest in successful outcomes from research grants and studentships as indicated in the Acknowledgements. No other conflicts of interest are disclosed.

## AUTHOR CONTRIBUTIONS

G.P., A.J.H. and J.A.K. designed and performed the calcium measurement assays. K.C. and C.H.R. designed, synthesised and analysed the chemicals. N.E. designed and performed the myography assays. O.V.P. designed and performed the PIEZO1 patch‐clamp experiments and J.A.K. the PIEZO2 patch‐clamp experiments. N.M., M.G.R., N.B. and A.B. designed the automated patch‐clamp experiments. N.M. and M.G.R. performed the automated patch‐clamp assays. T.S.F. and L.L. bred and maintained the genetically engineered mice. M.J.L. generated the cell lines. G.P., N.E., N.M. and O.V.P. made the figures. G.P. orchestrated the figure designs, data analysis and data transparency. C.H.R. generated the supplementary chemistry information. E.C.‐B. made intellectual contribution and performed the PIEZO2 and HeLa cell experiments with M.D. and F.B. G.P. and D.J.B. interpreted the data and wrote most of the manuscript with input from M.G.R., R.F., O.V.P., N.M., E.C.‐B. and M.D. D.J.B. and R.F. conceptualised the study, supervised the project team and generated funding.

## DECLARATION OF TRANSPARENCY AND SCIENTIFIC RIGOUR

This Declaration acknowledges that this paper adheres to the principles for transparent reporting and scientific rigour of preclinical research as stated in the *BJP* guidelines for Design & Analysis and Animal Experimentation and as recommended by funding agencies, publishers and other organisations engaged with supporting research.

## Supporting information


**Figure S1.** SI 1 Lack of agonism at TRPC5 channels.
**Figure S1.** SI 2. In‐house solubility comparisons for Yoda1, KC159 and KC289.
**Figure S2.** SI 1 Supporting data for Figure 2
**Figure S4.** SI 1 Supporting data for Figure 4
**Figure S6.** SI 1. Quantification of sustained current after application of KC157 and KC159.
**Figure S8.** SI 1 Effects of L‐NAME on vessel response to PE and resting basal tension
**Figure S8.** SI 2 All original traces for the data of Figure
**Figure S8.** SI 3 Dooku1 antagonises vasorelaxant effects of KC159 and KC289
**Figure S9.** SI 1 Example single experiment data in support of Figure 9
**Figure S9.** SI 2 Further analysis of data in Figure 9 – effects of long exposure
**Figure S10.** SI 1 Validation of transfection efficiency in HEK 293 and HeLa cells
**Figure S10.** SI 2 Detection of mechanically activated ionic current in HEK 293 cells overexpressing mouse PIEZO2
**Table S1.** Binding results for 30 targets.
**Table S2.** Physico‐chemical properties of Yoda1, KC159 and KC289.

## Data Availability

The data that support the findings of this study are available from the corresponding authors upon reasonable request. Some data may not be made available because of privacy or ethical restrictions. All original data are available in an Excel file. Unique laboratory materials created in the project are available on request (D.J.B. for biological materials and R.F. for chemicals).
