# Supplementary material for: Improved PIEZO1 agonism through 4‐benzoic acid modification of Yoda1
Source: Br J Pharmacol. 2023 Jan 31;180(16):2039–63. doi: 10.1111/bph.15996 (PMC10952572; doi:10.1111/bph.15996)
Supplement: Supplementary file 1 — Figure S1. SI 1 Lack of agonism at TRPC5 channels. Figure S1. SI 2. In‐house solubility comparisons for Yoda1, KC159 and KC289. Figure S2. SI 1 Supporting data for Figure 2 Figure S4. SI 1 Supporting data for Figure 4 Figure S6. SI 1. Quantification of sustained current after application of KC157 and KC159. Figure S8. SI 1 Effects of L‐NAME on vessel response to PE and resting basal tension Figure S8. SI 2 All original traces for the data of Figure Figure S8. SI 3 Dooku1 antagonises vasorelaxant effects of KC159 and KC289 Figure S9. SI 1 Example single experiment data in support of Figure 9 Figure S9. SI 2 Further analysis of data in Figure 9 – effects of long exposure Figure S10. SI 1 Validation of transfection efficiency in HEK 293 and HeLa cells Figure S10. SI 2 Detection of mechanically activated ionic current in HEK 293 cells overexpressing mouse PIEZO2 Table S1. Binding results for 30 targets. Table S2. Physico‐chemical properties of Yoda1, KC159 and KC289. [file BPH-180-2039-s001.pdf]

SUPPORTING INFORMATION (SI)

Improved PIEZO1 agonism through 4-benzoic acid modification of Yoda1

Gregory Parsonage[1†], Kevin Cuthbertson[2†], Naima Endesh[1], Nicoletta Murciano[3,4], Adam J. Hyman[1], Charlotte H. Revill[2], Oleksandr V. Povstyan[1], Eulashini Chuntharpursat-Bon[1], Marjolaine Debant[1], Melanie J. Ludlow[1], T. Simon Futers[1], Laeticia Lichtenstein[1], Jacob A. Kinsella[1], Fiona Bartoli[1], Maria Giustina Rotordam[3,4], Nadine Becker[3], Andrea Brüggemann[3], Richard Foster[2*], David J. Beech[1*]

[1]Leeds Institute of Cardiovascular and Metabolic Medicine, School of Medicine, University of Leeds, LS2 9JT, UK. [2]School of Chemistry, University of Leeds, LS2 9JT, UK. [3]Nanion Technologies GmbH, Ganghoferstr. 70A, DE - 80339 München, Germany. [4]Saarland University, Theoretical Medicine and Biosciences, Kirrbergerstr. 100, DE - 66424 Homburg, Germany.

*Addresses for correspondence: Professor D. J. Beech, Leeds Institute of Cardiovascular and Metabolic Medicine, LIGHT Building, Clarendon Way, School of Medicine, University of Leeds, Leeds LS2 9JT, UK. E-mail d.j.beech@leeds.ac.uk. Telephone +44 (0) 113 3434323. Dr R. Foster, School of Chemistry, University of Leeds, Leeds LS2 9JT, UK. E-mail r.foster@leeds.ac.uk. Telephone +44 (0) 113 3435759.

[†]Equal contributors

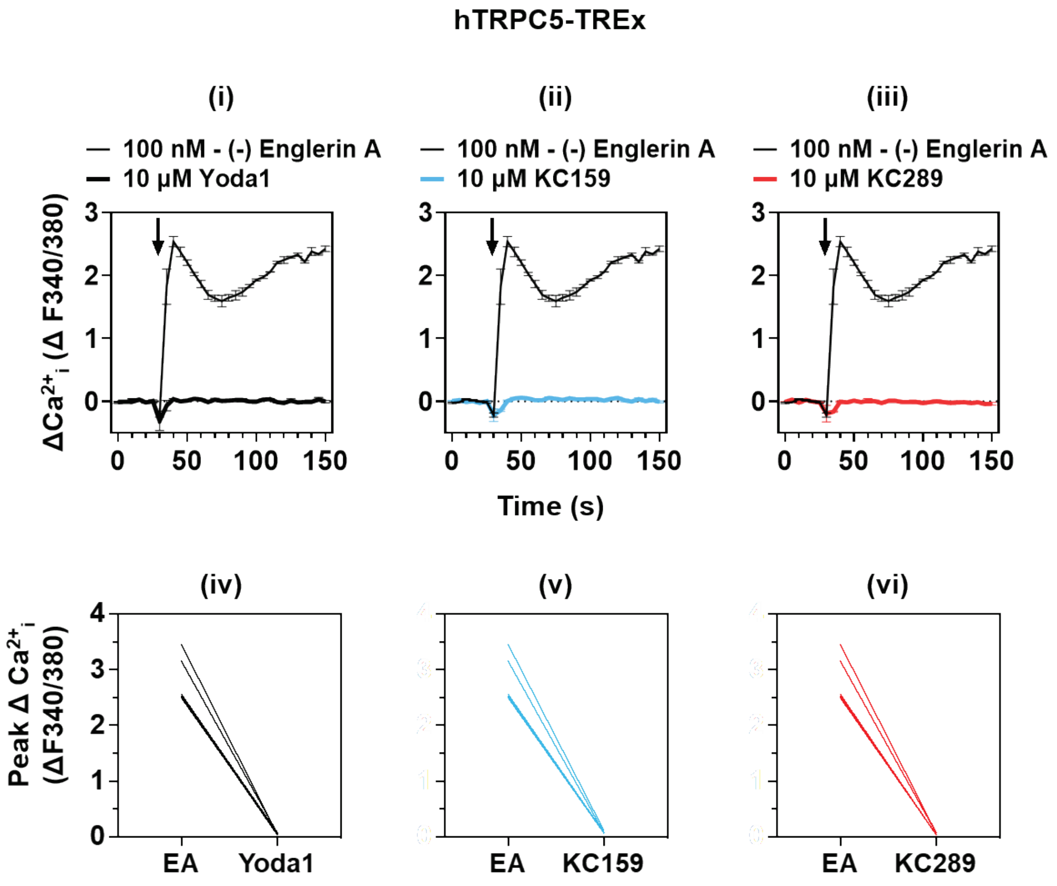

Figure S1. SI 1. Lack of agonism at TRPC5 channels.
(upper panel) Background-subtracted (Δ) intracellular Ca^{2+} measurements from a single experiment in which hTRPC5-TREx cells were acutely exposed to 10 µM (i) Yoda1, (ii) KC159, (iii) KC289. Arrows indicate the time at which the indicated compound was added to the cells, following a 30 s background read. The positive control stimulus (100 nM - (-) Englerin A is shown for comparison. Mean ± SEM values from 3 technical replicates are shown.
(lower panel) Paired, background-subtracted (Δ) peak intracellular Ca^{2+} measurement comparisons of hTRPC5-TREx cells treated with 100 nM (-)–Englerin A (EA) versus 10 µM (iv) Yoda1, (v) KC159, and (vi) KC289. Each plot shows mean peak values from independent experiments (n=5 for all compounds).

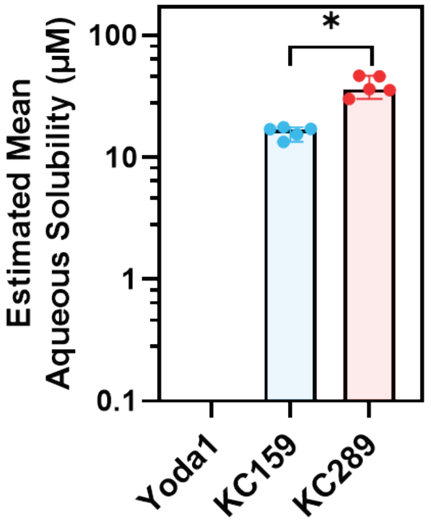

Figure S1. SI 2. In-house solubility comparisons for Yoda1, KC159 and KC289. Estimated solubility of Yoda1, KC159, and KC289 in universal aqueous buffer. Median values with data range from 5 independent experiments are shown (n=5). The lower limit of detection for the assay was 0.1 μM. Data for Yoda1 were below this limit, suggesting that Yoda1 was poorly soluble in these conditions. Results of a two-tailed Mann-Whitney rank comparison of the median values of the collated experiments are shown as: * $p < 0.05$.

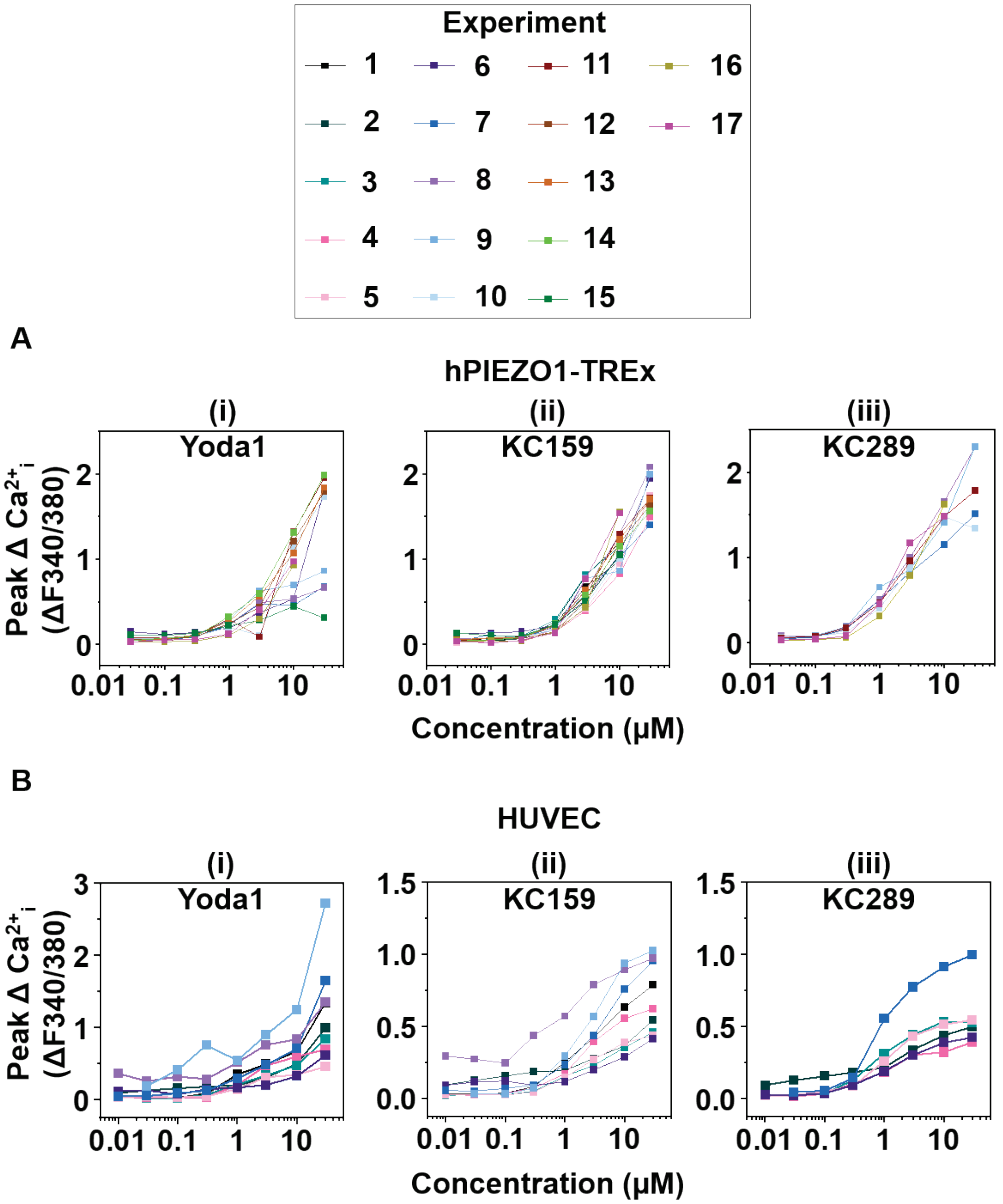

Figure S2. SI 1. Supporting data for Figure 2

(A) Background-subtracted (Δ) peak intracellular Ca^{2+} measurements from individual experiments in which hPIEZO1-TREx cells were exposed to the indicated concentrations of: (i) Yoda1 (n=12); (ii) KC159 (n=17); (iii) KC289 (n=7). Mean values from 2 to 5 technical replicates are shown for each of the individual experiments.

(B) Background-subtracted (Δ) peak intracellular Ca^{2+} measurements from individual experiments in which HUVECs were exposed to the indicated concentrations of: (i) Yoda1 (n=9); (ii) KC159 (n=9); (iii) KC289 (n=6). Mean values from 2 to 5 technical replicates are shown for each of the individual experiments.

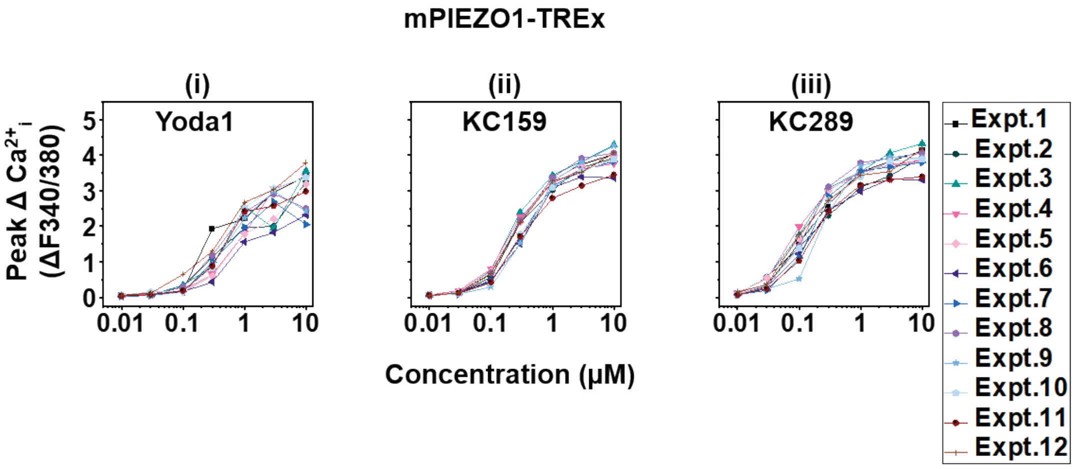

Figure S4. SI 1. Supporting data for Figure 4
Background-subtracted (Δ) peak intracellular Ca^{2+} measurements from individual experiments in which mPIEZO1-TREx cells were exposed to the indicated concentrations of; (i) Yoda1, (ii) KC159, (iii) KC289. Mean values from 2 or 3 technical replicates are shown for each of the individual experiments (n=12 for all compound tests).

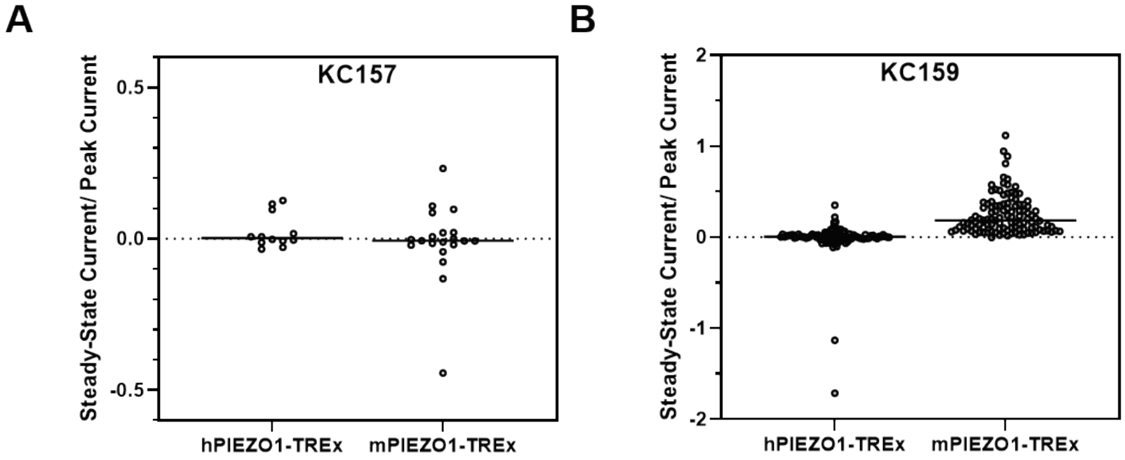

Figure S6. SI 1. Quantification of sustained current after application of KC157 and KC159.

Ratio of sustained current to peak current after application of (A) KC157 and (B) KC159 for mouse PIEZO1 and human PIEZO1. (A) hPIEZO1-TREx M-Stim + KC157 (n=12), mPIEZO1-TREx M-Stim + KC157 (n=20). (B) hPIEZO1-TREx M-Stim + KC159 (n=94), mPIEZO1-TREx M-Stim + KC159 (n=113). Bars indicate median values.

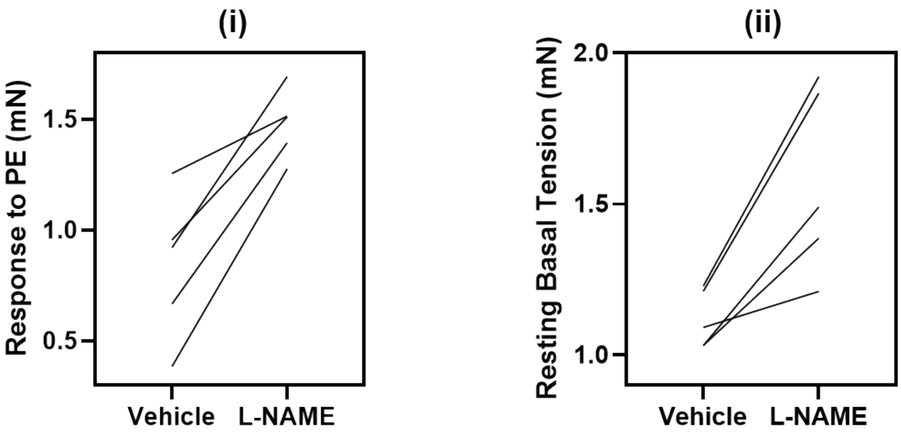

Figure S8. SI 1. Effects of L-NAME on vessel response to PE and resting basal tension Paired comparisons of (i) tension responses to PE and; (ii) isometric basal tension measurements before and after a 30-minute pre-incubation of vessels with 100 µM Nω-nitro-L-arginine methyl ester (L-NAME). Each plot shows values from independent experiments (n=5).

A **Control**

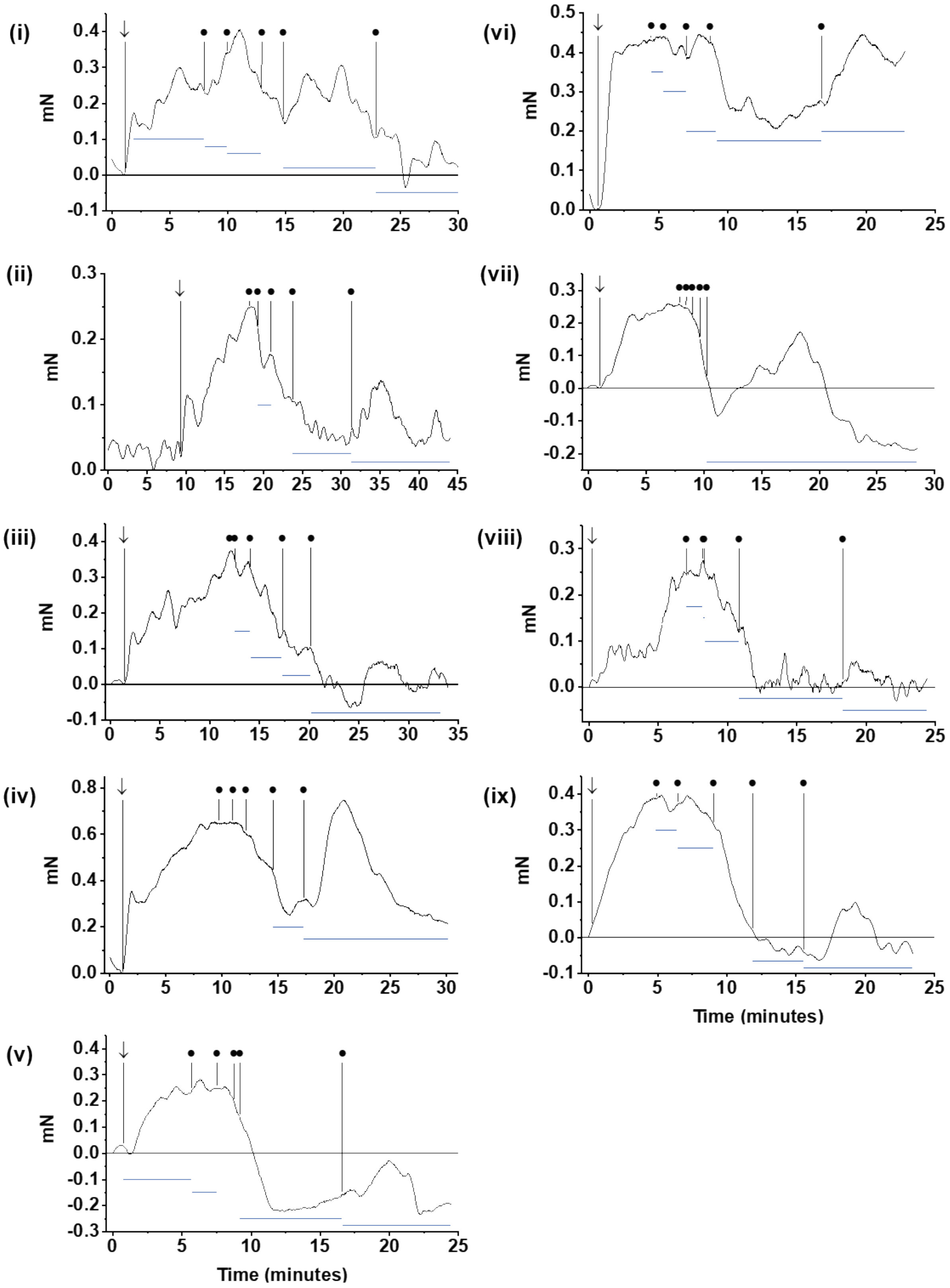

B **PIEZO1$^{\Delta EC}$**

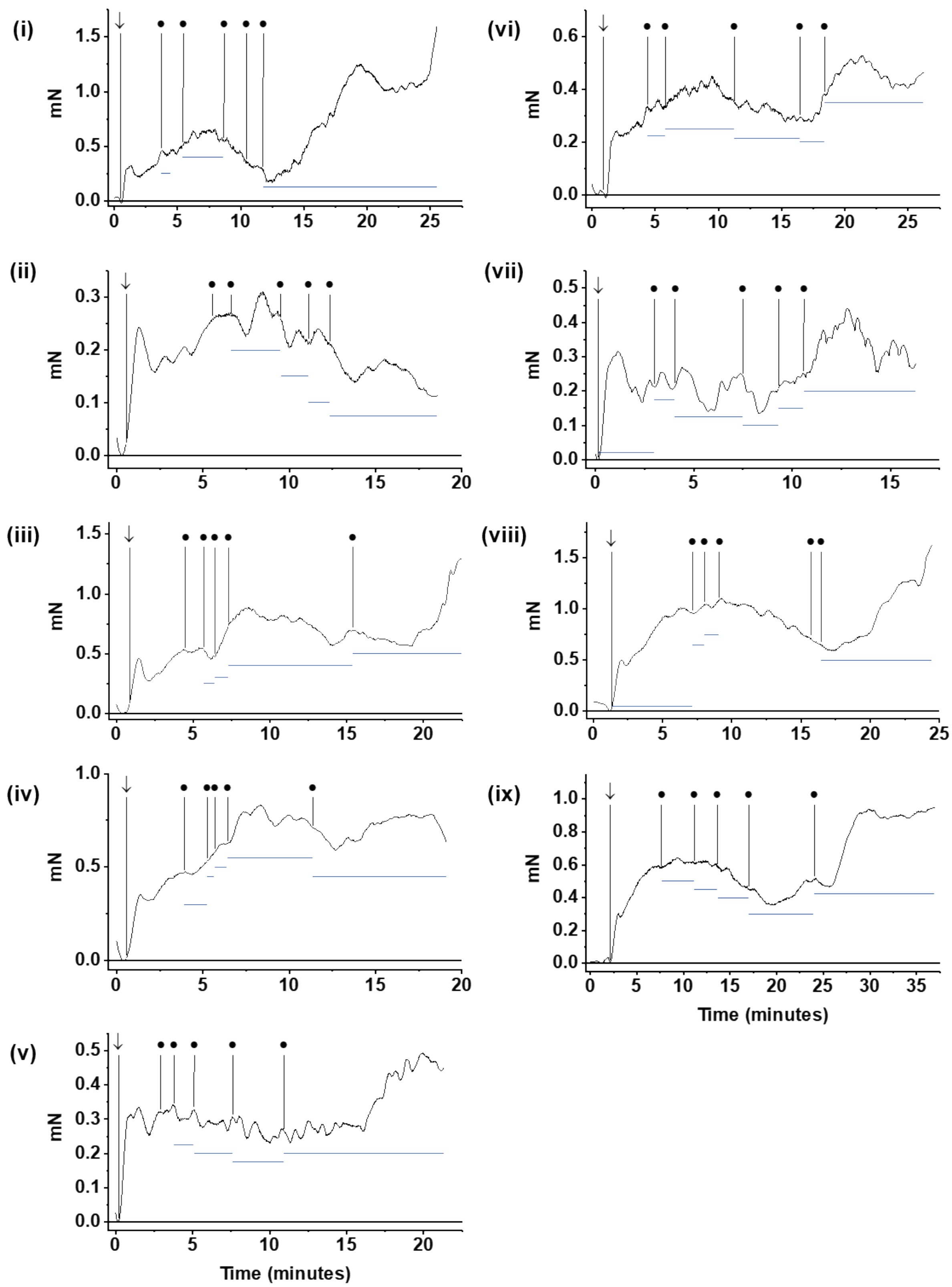

Figure S8. SI 2. All original traces for the data of Figure 8C.
Isometric tension traces from single experiments in which (A) (i-ix) control mouse, or (B) (i - ix) portal vein segments possessing intact endothelium were exposed to increasing concentrations (0.1, 0.3, 1, 3 and 10 μM) of KC289 after pre-constriction with 10 μM

phenylephrine (PE). The arrows indicate the addition of PE; dots indicate the sequential addition of rising concentrations of KC289. Blue lines annotate treatment periods in which spontaneous activity of the vessel made it difficult to determine if there were effects of the compound.

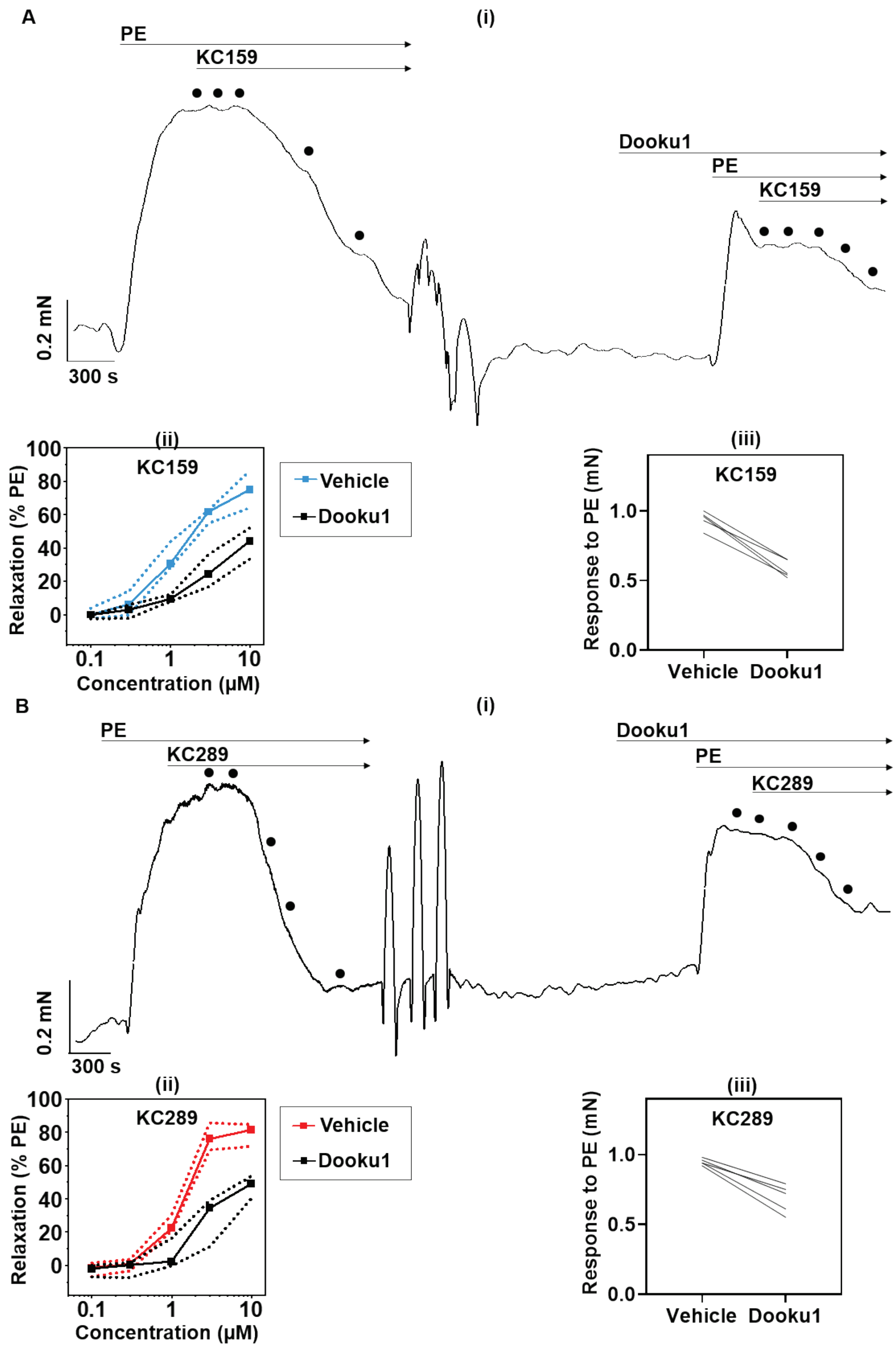

Figure S8. SI 3. Dooku1 antagonises vasorelaxant effects of KC159 and KC289

Isometric tension responses observed on addition of increasing concentrations of: (A) KC159 or; (B) KC289 before and after a 30-minute pre-incubation of the vessel with 10 μM Dooku1 or DMSO vehicle control. (A)(i) and (B)(i) show traces from individual experiments (n=1 each). The dots indicate the sequential addition of rising concentrations of compounds. (A)(ii) and (B)(ii) show corresponding dose-response data expressed as a % of the maximal PE-induced tension (n=5 for each compound). Median and range from collated experiments are shown. (A)(iii) and (B)(iii) show paired comparisons of vessel tension responses to PE. Each plot shows values from independent experiments (n=5).

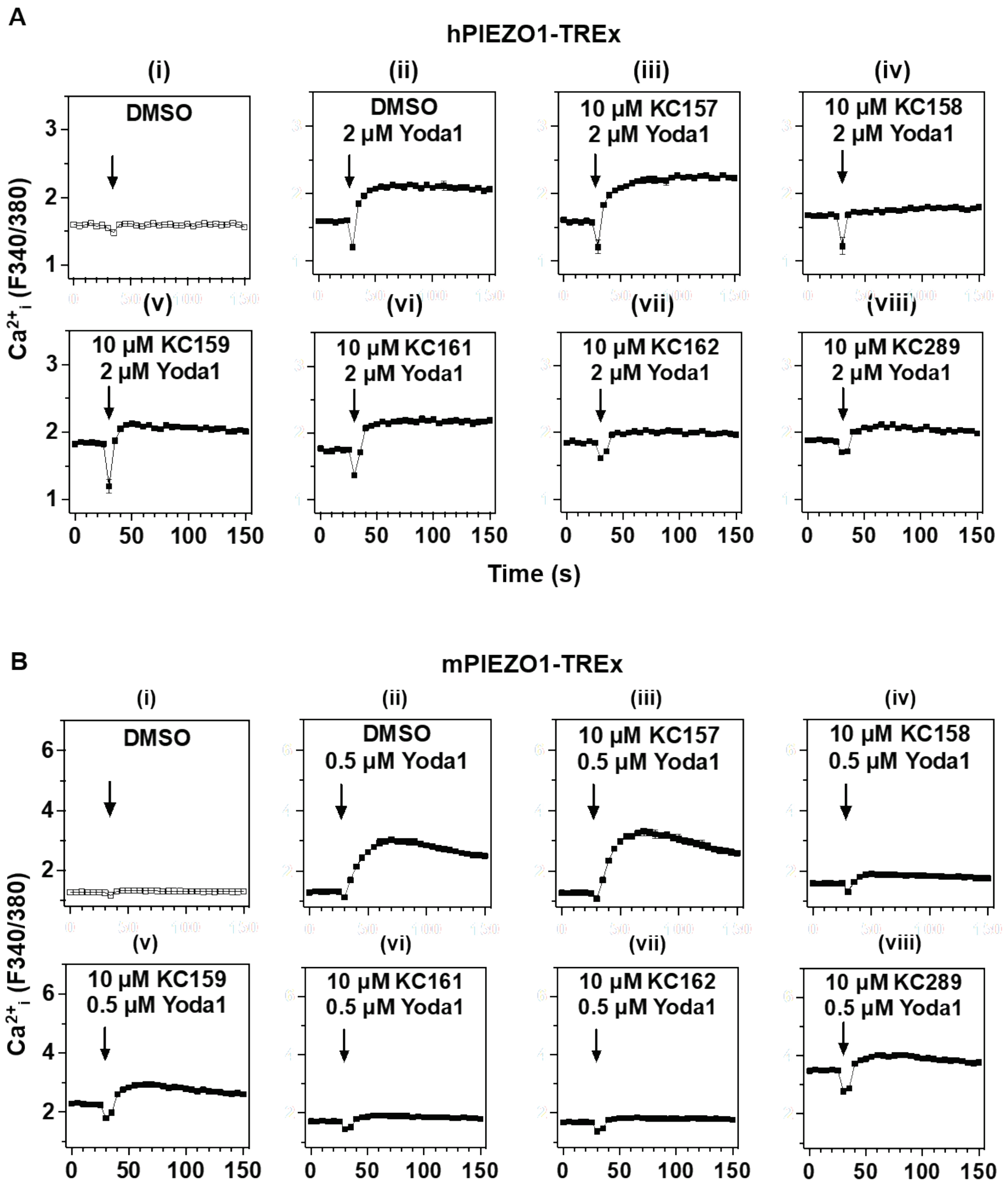

Figure S9. SI 1. Example single experiment data in support of Figure 9
Intracellular Ca^{2+} measurements from single experiments (n=1) in which; (A) hPIEZO1-TREx and; (B) mPIEZO1-TREx cells were pretreated with; (i - ii) DMSO vehicle, or 10 µM; (iii) KC157, (iv) KC158, (v) KC159, (vi) KC161, (vii) KC162, (viii) KC289 for 30 minutes before stimulation with the indicated concentration of Yoda1. Arrows indicate the time at which Yoda1 was added to the cells, following a 30 s background read. Mean ± SEM values from between 3 and 5 technical replicates are shown.

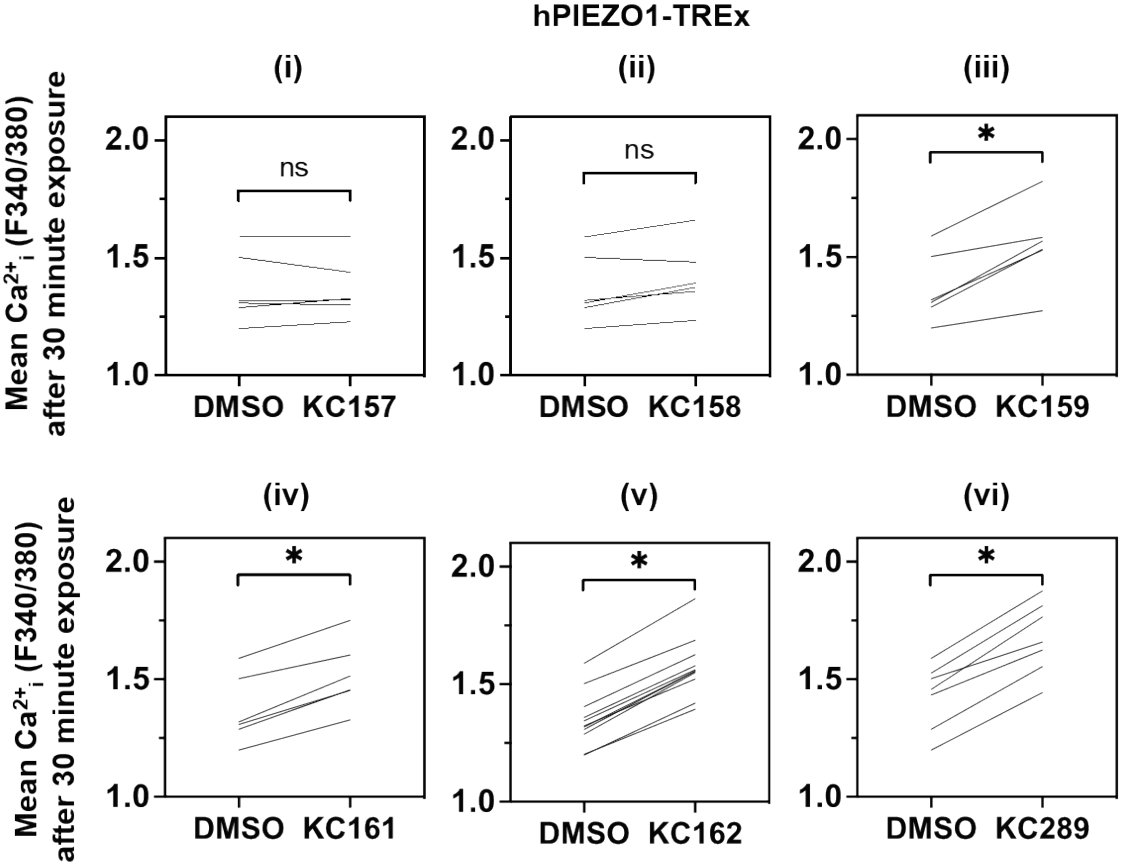

Figure 9. SI 2. Further analysis of data in Figure 9 – effects of long exposure
Paired mean intracellular Ca^{2+} measurement comparisons of hPIEZO1-TREx treated with 10 μM indicated compound for 30 minutes. Each plot shows mean values from independent experiments. Independent experiment numbers: (i) KC157, n=6; (ii) KC158, n=6; (iii) KC159, n=6; (iv) KC161, n=6; (v) KC162, n= 11; (vi) KC289, n=7. Paired sample Wilcoxon signed rank test results comparing the median values of the collated experiments are shown as: n.s ($p > 0.05$), * $p < 0.05$.

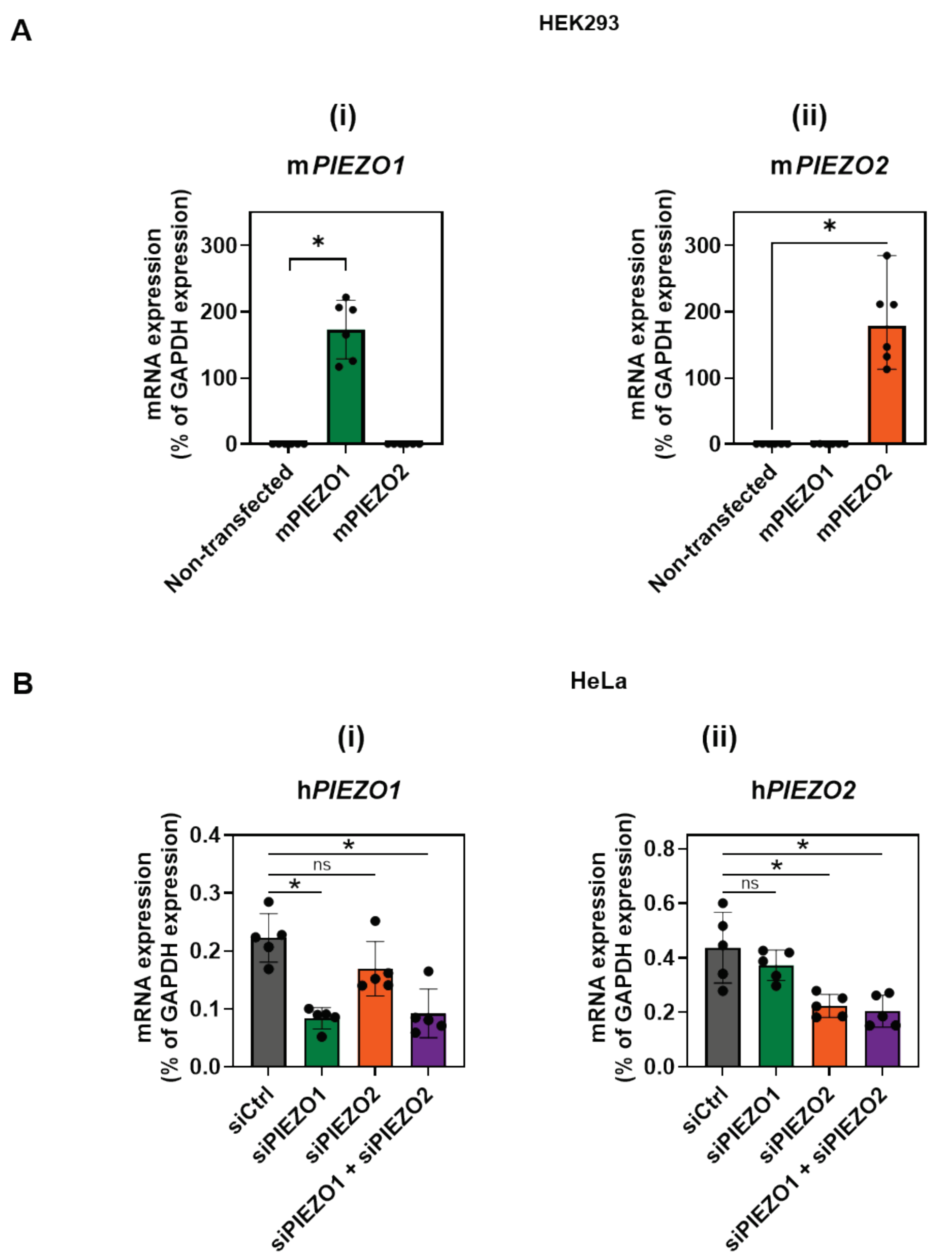

Figure S10. SI 1. Validation of transfection efficiency in HEK 293 and HeLa cells
(A) mRNA abundance of (i) m*PIEZO1* and (ii) m*PIEZO2* (n=5) transcripts relative to h*GAPDH* mRNA in HEK 293 cells overexpressing mPIEZO1, mPIEZO2 or neither ("non-transfected cells"). (B) mRNA abundance of (i) h*PIEZO1* or (ii) h*PIEZO2* transcripts (n=5) relative to h*GAPDH* expression in HeLa cells after siRNA mediated knockdown of hPIEZO1 (siPIEZO1), hPIEZO2 (siPIEZO2), both (siPIEZO1 + siPIEZO2) or transfected with a non-targeting (Control, Ctrl) siRNA (siCtrl). Data expressed as Mean ± SD except in Aii, which is Median with Range because the data could not be confirmed as normally distributed. One way ANOVA followed by a Tukey's post-hoc test for multiple comparison was performed on the mean values of the collated experiments (Ai, Bi and Bii) and Friedmann test followed by a Dunn's post-hoc test for multiple comparison for (Aii): n.s ($p > 0.05$), * $p < 0.05$ for comparison against non-transfected and siCtrl only.

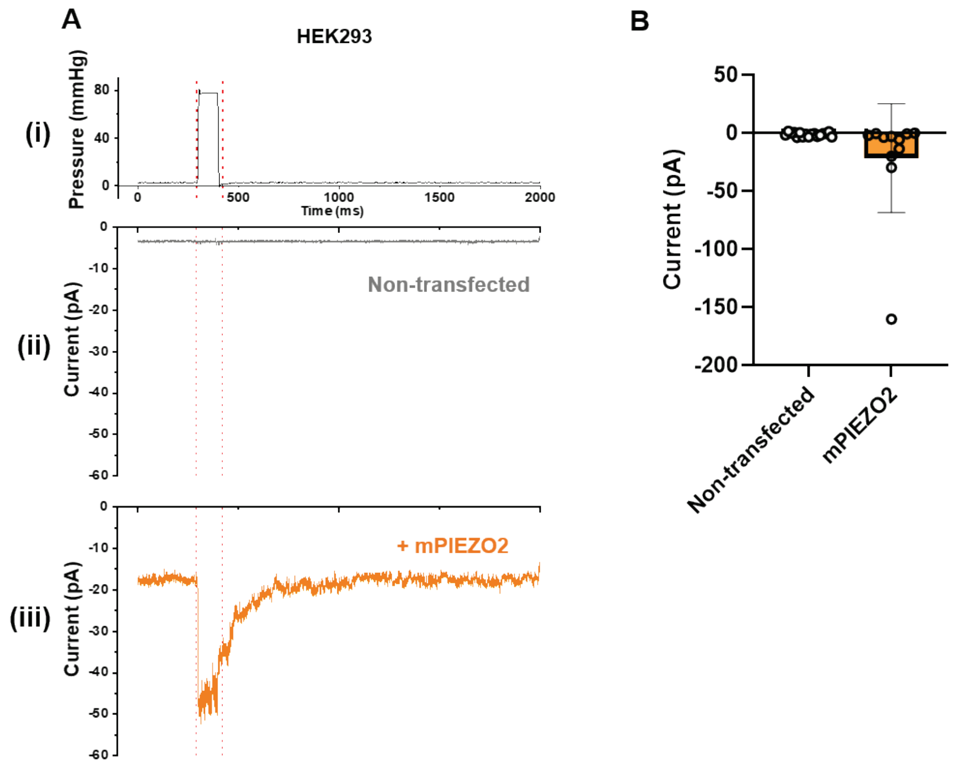

Figure S10. SI 2. Detection of mechanically activated ionic current in HEK 293 cells overexpressing mouse PIEZO2

(A) Example (n=1) outside-out patch current recordings showing the response to (i) 100-ms 75 mmHg positive pressure pulse for (ii) non-transfected HEK 293 cells and (iii) HEK 293 cells overexpressing mPIEZO2. Traces in (ii) and (iii) are average currents from 3 consecutive pressure pulses. (B) Mean ± SD for peak inward currents activated by the pressure pulse as exemplified in (A): non-transfected cells (N=17 independent recordings) and HEK 293 cells overexpressing mPIEZO2 (N=11 independent recordings).

Assay Name	Gene / Subfamily Name	Binding Assay Type	Catalogue #	Organism	% Inhibition
Acetylcholine M2 (Muscarinic)	CHRM2	GPCR (Antagonist Radioligand)	252710	Human	10
Acetylcholine M3 (Muscarinic)	CHRM3	GPCR (Antagonist Radioligand)	252810	Human	1
Adenosine A1	ADORA1	GPCR (Antagonist Radioligand)	200510	Human	-4
**Adenosine A2A**	**ADORA2A**	**GPCR (Agonist Radioligand)**	**200610**	**Human**	**40**
Adrenoceptor alpha1A	ADRA1A	GPCR (Antagonist Radioligand)	203100	Rat	3
Adrenoceptor alpha1B	ADRA1B	GPCR (Antagonist Radioligand)	203200	Rat	6
Adrenoceptor alpha2A	ADRA2A	GPCR (Antagonist Radioligand)	203630	Human	14
Adrenoceptor beta1	ADRB1	GPCR (Antagonist Radioligand)	204010	Human	11
Adrenoceptor beta2	ADRB2	GPCR (Antagonist Radioligand)	204110	Human	-2
Cav1.2 (L-type)	CACNA1C	Calcium Ion Channel (Dihydropyridine Site)	214600	Rat	20
CB1 Human Cannabinoid	CNR	GPCR (Antagonist Radioligand)	217050	Human	5
Dopamine D1	DRD1	GPCR (Antagonist Radioligand)	219500	Human	9
Dopamine D2S	DRD2	GPCR (Antagonist Radioligand)	219700	Human	1
GABA A	GABAA	(Non-Selective) Ion Channel [3H] Muscimol (Agonist Radioligand)	226500	Rat	12
GABA A	GABAA	(Non-Selective) Ion Channel [3H] Flunitrazepam (Agonist Radioligand)	226600	Rat	1
Glutamate	Ionotropic	(Non-Selective) Ion Channel [3H] TCP	233000	Rat	0
Histamine H1	HRH1	GPCR (Antagonist Radioligand)	239610	Human	-7
Imidazoline I2	Imidazoline	Central (Antagonist Radioligand)	241000	Rat	13
mu Opioid	OPRM1	GPCR (Antagonist Radioligand)	260410	Human	-10
nAChR (alpha1)	CHRNA1	Ion Channel, Bungarotoxin (Antagonist Radioligand)	258700	Human	-3
nAChR Nicotinic Acetylcholine	-	Ion Channel Binding, Epibatidine (Antagonist Radioligand binding)	258590	Human	-5
Norepinephrine Transporter	SLC6A2	Transporter (Antagonist Radioligand)	204410	Human	-9
PDE	Phosphodiesterase	(Non-Selective) [3H] Rolipram (Antagonist Radioligand)	270000	Rat	5
Phorbol Ester	-	Phorbol Ester	264500	Mouse	9
Potassium Channel (hERG)	KCNH2	Potassium Ion Channel [3H] Astemizole (Antagonist Radioligand)	265900	Human	3
Potassium Channel (KATP)	KCNJ11	Potassium Ion Channel (Antagonist Radioligand)	265600	Hamster	0
**Prostanoid EP4**	**PTGER4**	**GPCR (Agonist Radioligand)**	**268420**	**Human**	**63**
Serotonin (5-HT2B)	HTR2B	GPCR [3H]LSD (Agonist Radioligand)	271700	Human	-1
Sigma 1	SIGMAR1	Non-Selective (Antagonist Radioligand)	278110	Human	2
Sodium Ion Channel	Sodium Ion Channel	(Non-Selective) Sodium Ion Channel [3H] Batrachotoxinin (Site 2)	279510	Rat	-3

Table S1. Binding results for 30 targets.

Data are for KC289 at 5 μM. 'Phorbol ester' is a binding assay that targets protein kinase Cs and potentially other proteins. Data were produced by Eurofins Scientific (https://www.eurofins.co.uk/).

Assay	Repeat	Yoda1 Values	Yoda1 Mean	KC159 Values	KC159 Mean	KC289 Values	KC289 Mean
**Kinetic solubility (µM) (0.1 M phosphate buffer, pH 7.4)**	1<br>2	0.2<br>3.8	**2**	76.6<br>1.5	**39.1**	17.8<br>10.2	**14**
**Thermodynamic solubility (µM) (0.1 M phosphate buffer, pH 7.4)**	1<br>2	0.44<br>0.3	**0.37**	11.9<br>7.7	**9.8**	46<br>8.9	**27.5**
**Mouse Microsomal $t_{1/2}$ (min)**	1<br>2	1.0<br>1.2	**1.1**	37.2<br>21.9	**29.6**	31.8<br>17.3	**24.6**
**Mouse Plasma Protein Binding (% Fraction Unbound)**	1<br>2	<0.1<br>0.1	**<0.1**	0.4<br>0.8	**0.6**	0.5<br>0.8	**0.65**
**Mouse plasma stability (% compound remaining after 2 hours)**	1<br>2<br>3	nd<br>119<br>115	**117**	69<br>96<br>92	**85.7 ± 14.6 (S.D.)**	125<br>110<br>98	**111 ± 13.5 (S.D.)**

Table S2. Physico-chemical properties of Yoda1, KC159 and KC289.
Data were produced by Malvern PanAnalytical (https://www.malvernpanalytical.com/).

Chemistry SI

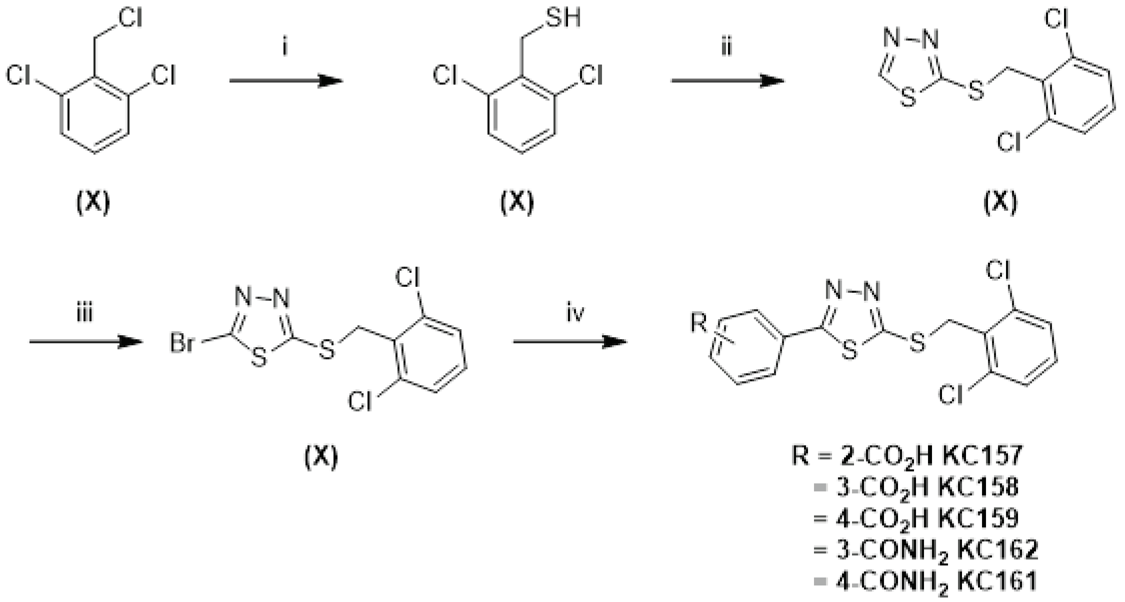

Scheme 1: i) a) thiourea (1.1 eq.), EtOH, reflux, 3h b) NaOH, EtOH, reflux, N_2, 3 h c) HCl (98%) ii) 2-bromo 1,3,4-thiadiazole (1.0 eq.), K_2CO_3 (1.2 eq.), DMF, 90 °C, 18 h (57%) iii) NBS (1.4 eq.), DCM, reflux, N_2, 48 h. (78%) iv) Appropriate boronic acid (1.0-3.5 eq.), K_2CO_3 (4.0 eq.), $Pd(PPh_3)_4$ (0.1-2 eq.) dioxane, H_2O, 90 °C, N_2, 2-24 h (7-68%)

Materials

All purchased chemicals and solvents were used without further purification unless otherwise stated. All compounds were at least 95% pure by ^1H NMR.

Physical Methods

^1H Nuclear Magnetic Resonance spectra were recorded at 500 MHz using a Bruker DRX 500 instrument or at 400 MHz using a Bruker DPX 400. ^1H spectra are referenced based on the residual proton in the solvent (*e.g.* the $CHCl_3$, 0.01 % in 99.99 % $CDCl_3$). Coupling constants (J) are reported to the nearest 0.1 Hz. ^{13}C NMR spectra were recorded at 125 MHz on 500 MHz spectrometers or at 100 MHz on 400 MHz spectrometers. HRMS was performed on a Burker Daltonics micrOTOF using positive electrospray ionisation (ES+). Automated column chromatography (ACC) was carried out using a Biotage Isolera Four EXP with Spektra.

General Procedure A

The desired aromatic halide (0.28 mmol, 1.0 eq. the desired boronic acid (0.28-0.98 mmol, 1-3.5 eq) and K_2CO_3 (1.12 mmol, 4.0 eq.) were dissolved in dioxane (2 mL) and H_2O (2 mL) then degassed with N_2 for 30 minutes. $Pd(PPh_3)_4$ (0.04 mmol, 0.15 eq.) was then added and the reaction was then heated to 90 °C for 2-24 h. Upon completion, the reaction was diluted with H_2O (20 mL), extracted with DCM (3 × 10 mL), dried over Na_2SO_3, filtered and reduced *in vacuo* to afford the crude or pure product.

2,6-dichlorobenzyl thiol (X)

2,6-dichlorobenzyl chloride (3.00 g 16.40 mmol) and thiourea (1.38 g, 18.40 mmol) were dissolved in EtOH (36 mL) and heated to reflux for 3 h. The solution was cooled to room temperature and dissolved in 2 M aq. NaOH (30 mL) and EtOH (10 mL) and the reaction was

refluxed for a further 3 hours. The solution was then cooled, quenched with 1 M HCl (60 mL), reduced *in vacuo*, and extracted with EtOAc (3 × 20 mL). The combined organic layers were then washed with sat. NaCl solution (2 × 20 mL), dried over Na_2SO_4 and evaporated to dryness to afford an off-white crystalline solid without further need for purification (2.90 g, 15.00 mmol, 98%). Rf 0.7 (9:1 Petroleum ether (40-60 °C):EtOAc (v/v)); δ_H (500 MHz, $CDCl_3$): 7.33 (2H, d, *J* = 8.0 Hz, benzyl 3-H), 7.15 (1H, t, *J* = 8.0 Hz, benzyl 4-H), 4.02 (2H, d, *J* = 8.5 Hz, benzyl CH_2), 2.12 (1H, t, *J* = 8.5 Hz, SH); δ_C (125 MHz, $CDCl_3$): 137.3 (benzyl 1-C), 134.6 (benzyl 2-C), 128.5 (benzyl 4-C), 128.5 (benzyl 3-C), 24.4 (benzyl CH_2).

2-((2,6-dichlorobenzyl)thio)-1,3,4-thiadiazole (X)

2,6-dichlorobenzyl thiol (2.85 g, 14.77 mmol), 2-bromo-1,3,4-thiadiazole (2.43 g, 14.77 mmol),& K_2CO_3 (2.38 g, 17.72 mmol) were dissolved in DMF (10 mL) and heated to 90 °C for 18 h. The reaction was diluted with H_2O (100 mL), extracted with EtOAc (3 × 40 mL) and the organic layers combined. These were washed with brine (3 × 40 mL), 10% LiCl (3 × 40 mL), dried over $MgSO_4$, filtered and reduced *in vacuo* to give brown residue (4.33 g). This was purified by ACC (0-30% EtOAc in petroleum ether (40-60 °C)) to afford white crystalline solid (2.33 g, 8.43 mmol, 57%) Rf 0.60 (7:3 Petroleum ether (40-60 °C):EtOAc (v/v)); δ_H (400 MHz, $CDCl_3$): 8.99 (1H, s, thiadiazole 5-H), 7.27 (2H, d, *J* = 8.5 Hz, benzyl 3-H), 7.13 (1H, t, *J* = 8.5 H, benzyl 4-H), 4.89 (2H, s, benzyl CH_2); δ_C (100 MHz, $CDCl_3$): 164.7 (thiadiazole 2-C), 152.1 (thiadiazole 5-C), 136.3 (benzyl 2-C), 131.7 (benzyl 1-C), 129.8 (benzyl 4-C), 128.5 (benzyl 3-C), 34.73 (benzyl CH_2); m/z ES+ Found MNa^+ 298.9235, $C_9H_6Cl_2N_2S_2$ requires MNa^+ 298.9242.

2-Bromo-5-((2,6-dichlorobenzyl)thio)-1,3,4-thiadiazole

2-((2,6-dichlorobenzyl)thio)-1,3,4-thiadiazole (2.33 g, 8.43 mmol), & N-bromosuccinimide (2.10 g, 11.80 mmol) were dissolved in DCM (10 mL) and refluxed for 48 h. The reaction was then cooled, quenched with sat. $Na_2S_2O_3$ (20 mL), partitioned and the aqueous extracted with DCM (2 × 15 mL). The organic layers were combined, dried over $MgSO_4$, filtered and reduced *in vacuo* to give an orange, oily crystals (3.15 g). This was purified by ACC (0-30% EtOAc in Petroleum ether (40-60 °C)) to afford a white crystalline solid (2.80 g, 7.87 mmol, 78 %) Rf 0.80 (7:3 Petroleum ether (40-60 °C):EtOAc (v/v)); δ_H (400 MHz, $CDCl_3$): 7.37 (2H, d, *J* = 8 Hz, benzyl 3-H), 7.24 (1H, dd, *J* = 8.5 & 7.5 Hz, benzyl 4-H), 4.92 (2H, s benzyl CH_2); δ_C (100 MHz, $CDCl_3$): 168.1 (thiadiazole 5-C), 138.1 (thiadiazole 2-C), 136.3 (benzyl 2-C), 131.5 (benzyl 1-C), 129.9 (benzyl 4-C), 128.6 (benzyl 3-C), 34.6 (benzyl CH_2); m/z ES+ Found MH^+ 356.8462, $C_9H_5BrCl_2N_4S_2$ requires MH^+ 356.8512.

2-(5-((2,6-Dichlorobenzyl)thio)-1,3,4-thiadiazol-2-yl)benzoic acid (KC157)

General procedure A was followed using 2-bromo-5-((2,6-dichlorobenzyl)thio)-1,3,4-thiadiazole (100 mg, 0.28 mmol), 2-(methoxycarbonyl)phenylboronic acid (43 mg, 0.28 mmol), K_2CO_3 (155 mg, 1.12 mmol), $Pd(PPh_3)_4$ (35 mg, 0.03 mmol), dioxane (2 mL) and water (2 mL) to afford a crude orange solid. This was triturated with DCM to afford a yellow powder (16 mg, 0.04 mmol, 14%). δ_H (400 MHz, D_6-DMSO): 7.91-7.88 (1H, m, benzoatyl 6-H), 7.73-7.67 (3H, m, benzoatyl 3, 4 & 5-H), 7.56 (2H, d, *J* = 8.0 Hz, benzyl 3-H), 7.42 (1H, t, *J* = 8.0 Hz, benzyl 4-H), 4.85 (2H, s, benzyl CH_2); δ_C (100 MHz, D_6-DMSO): 168.4 (carbonyl C), 167.9 (thiadiazolyl 2-C), 164.7 (thiadiazolyl 5-C), 135.7 (benzoatyl 6-C), 133.1 (benzoatyl 1 or 2-C or benzyl 1-C), 132.1 (benzoatyl 3, 4 or 5-C), 131.9 (benzoatyl 1 or 2-C or benzyl 1-C), 131.6 (benzoatyl 3, 4 or 5-C) 131.4 (benzyl 4-C), 131.3 (benzoatyl 3, 4 or 5-C), 130.3 (benzyl 2-C), 129.4 (benzyl 3-C), 128.9 (benzoatyl 3, 4 or 5-C), 34.9 (benzyl CH_2); m/z ES+ Found MH^+ 396.9635, $C_{16}H_{10}Cl_2N_2O_2S_s$ requires MH^+ 396.9639.

3-(5-((2,6-Dichlorobenzyl)thio)-1,3,4-thiadiazol-2-yl)benzoic acid (KC158)

General procedure A was followed using 2-bromo-5-((2,6-dichlorobenzyl)thio)-1,3,4-thiadiazole (100 mg, 0.28 mmol), 3-(methoxycarbonyl)phenylboronic acid (43 mg, 0.28 mmol), K_2CO_3 (155 mg, 1.12 mmol), $Pd(PPh_3)_4$ (35 mg, 0.03 mmol), dioxane (2 mL) and water (2 mL) to afford a crude orange solid. This was triturated with DCM to afford a yellow powder (6 mg, 0.02 mmol, 7%) δ_H (400 MHz, D_6-DMSO): 8.43 (1H, s. benzoatyl 2-H), 8.17 (1H, d, *J* = 8.0 Hz, benzoatyl 4 or 6-H), 8.13 (1H, d, *J* = 8.0 Hz, benzoatyl 4 or 6-H), 7.71 (1H, ap. t, *J* = 8.0 Hz, benzoatyl 5-H), 7.57 (2H, d, *J* = 8.0 Hz, benzyl 3-H), 743 (1H, t, 8.0 Hz, benzyl 4-H), 4.87 (2H, s, benzyl CH_2); δ_C (100 MHz, D_6-DMSO): 169.0 (carbonyl C), 166.9 (thiadiazolyl 2-C), 164.2 (thiadiazolyl 5-C), 135.7 (benzyl 2-C), 132.4 (benzyl 1-C or benzoatyl 3-C), 132.2 (benzoatyl 4 or 6-C), 132.0 (benzoatyl 4 or 6-C), 131.4 (benzyl 4-C), 130.5 (benzoatyl 5-C), 130.0 (benzyl 1-C or benzoatyl 3-C), 129.4 (benzyl 3-C), 128.3 (benzoatyl 2-C), 35.1 (benzyl CH_2); m/z ES+ Found MH^+ $C_{16}H_{10}Cl_2N_2O_2S_s$ requires MH^+ 396.9639.

4-(5-(2,6-Dichlorobenzyl)thio)-1,3,4-thiadiazol-2-yl)benzoic acid (KC159)

General procedure A was followed using 2-bromo-5-((2,6-dichlorobenzyl)thio)-1,3,4-thiadiazole (100 mg, 0.28 mmol), 4-(methoxycarbonyl)phenylboronic acid (43 mg, 0.28 mmol), K_2CO_3 (155 mg, 1.12 mmol), $Pd(PPh_3)_4$ (35 mg, 0.03 mmol), dioxane (2 mL) and water (2 mL) to afford a crude orange solid. This was triturated with DCM to afford an orange powder (77 mg, 0.19 mmol, 68%) δ_H (400 MHz, D_6-DMSO): 8.09-8.04 (4H, m, benzoate 2 & 3-H), 7.56 (2H, d *J* = 8.0 Hz, benzyl 3-H), 7.43 (1H, t, *J* = 8.0 Hz, benzyl 4-H), 4.88 (2H, s, benzyl CH_2);δ_C (100 MHz, D_6-DMSO): 168.8 (thiadiazolyl 2-C), 167.0 (thiadiazolyl 5-C), 164.8 (carbonyl C), 135.7 (benzyl 2-C), 133.6 (benzoatyl 1 or 4-C), 133.3 (benzoatyl 1 or 4-C), 132.0 (benzyl 1-C), 131.4 (benzyl 4-C), 130.1 (benzoatyl 2 or 3-C), 129.4 (benzyl 3-C) 128.3 (benzoatyl 2 or 3-C), 35.1 (benzyl CH_2); m/z ES+ Found MH^+ 396.9627, $C_{16}H_{10}Cl_2N_2O_2S_s$ requires MH^+ 396.9639.

4-(5-((2,6-Dichlorobenzyl)thio)-1,3,4-thiadiazol-2-yl)benzamide (KC161)

General procedure A was followed using 2-bromo-5-((2,6-dichlorobenzyl)thio)-1,3,4-thiadiazole (100 mg, 0.28 mmol), 4-(aminocarbonyl)-phenylboronic acid (46 mg, 0.28 mmol), K_2CO_3 (155 mg, 1.12 mmol), $Pd(PPh_3)_4$ (35 mg, 0.03 mmol), dioxane (2 mL) and water (2 mL) to afford a crude brown solid. This was purified by triturations with EtOAc and then DCM to afford a yellow solid (25 mg, 0.06 mmol, 21%). δ_H (400 MHz, D_6-DMSO): 8.03 (4H, bs, benzamidyl 2 & 3-H), 7.57 (2H, d, *J* = 8.0 Hz, benzyl 3-H), 7.43 (1H, t, *J* = 8.0 Hz, benzyl 4-H), 4.87 (2H, s, benzyl CH_2); δ_C (100 MHz, D_6-DMSO): 169.0 (carbonyl C), 167.4 (thiadiazolyl 2-C), 164.3 (thiadiazolyl 5-C), 137.2 (benzamidyl 1 or 4-C), 135.7 (benzyl 2-C), 132.0 (benzyl 1-C or benzamide 1 or 4-C), 131.9 (benzyl 1-C or benzamidyl 1 or 4-C), 131.4 (benzyl 4-C), 129.4 (benzamidyl 2 or 3-C), 129.0 (benzyl 3-C), 128.0 (benzamidyl 2 or 3-C), 35.1 (benzyl CH_2; m/z ES+ Found MNa^+ 417.9608, $C_{16}H_{11}Cl_2N_3OS_2$ requires MNa^+ 417.9612.

3-(5-((2,6-Dichlorobenzyl)thio)-1,3,4-thiadiazol-2-yl)benzamide (KC162)

General procedure A was followed using 2-bromo-5-((2,6-dichlorobenzyl)thio)-1,3,4-thiadiazole (100 mg, 0.28 mmol), 3-(aminocarbonyl)-phenylboronic acid (46 mg, 0.28 mmol), K_2CO_3 (155 mg, 1.12 mmol), $Pd(PPh_3)_4$ (35 mg, 0.03 mmol), dioxane (2 mL) and water (2 mL) to afford a crude brown solid. This was purified by triturations with EtOAc and then DCM to afford a yellow solid (44 mg, 0.10 mmol, 36%). δ_H (400 MHz, D_6-DMSO): 8.39 (1H, s, benzamidyl 2-H), 8.10 (1H, d, *J* = 8.0 Hz, benzamidyl 4 or 6-H), 8.07 (1H, d, *J* = 8.0 Hz, benzamidyl 4 or 6-H), 7.99 (1H, ap. t, *J* = 7.5 Hz, benzamidyl 5-H), 7.56 (2H, d, *J* = 8.0 Hz, benzyl 3-H), 7.43 (1H, t, *J* = 8.0 Hz, benzyl 4-H), 4.86 (2H, s, benzyl CH_2); δ_C (100 MHz, D_6-DMSO):169.3 (carbonyl C), 167.3 (thiaidazolyl 2-C), 164.0 (thiadiazolyl 5-C), 136.9 (benzamidyl 3-C), 135.6 (benzyl 2-C), 132.0 (benzamidyl 1-C), 131.3 (benzyl 4-C), 130.8 (benzamidyl 4 or 6-C), 130.6 (benzamidyl 4 or 6-C), 130.1 (benzamidyl 5-C), 129.8 (benzyl 1-

C), 129.4 (benzyl 3-C), 127.1 benzamidyl 2-C), 35.1 (benzyl CH_2); m/z ES+ Found MNa^+ 417.9609, $C_{16}H_{11}Cl_2N_3OS_2$ requires MNa^+ 417.9612.

Potassium 4-(5-((2,6-dichlorobenzyl)thio)-1,3,4-thiadiazol-2-yl)benzoate (KC289)

4-(5-((2,6-dichlorobenzyl)thio)-1,3,4-thiadiazol-2-yl)benzoic acid (20 mg, 0.05 mmol) and KOH (2.81 mg, 0.05 mmol) were dissolved in MeOH (3 mL) and heated to 60 °C for 18 h. The reaction was then reduced under pressure to give a white solid without further need for purification (20.92 mg, 0.05 mmol, 100%). δ_H (500 MHz, D_6-DMSO): 7.97 (2H, d, *J* = 8.0 Hz, benzoatyl 2-H), 7.85 (2H, d, *J* = 8.0 Hz, benzoatyl 3-H), 7.56 (2H, d, *J* = 8.0 Hz, benzyl 3-H), 7.43 (1H, dd, *J* = 8.5 & 7.5 Hz, benzyl 4-H), 4.85 (2H, s, benzyl CH_2); δ_C (125 MHz, D_6-DMSO): 170.0 (carbonyl-C), 167.5 (thiadiazolyl 2-C), 162.7 (thiadiazolyl 5-C), 135.6 (benzyl 2-C), 132.1 (benzyl 1-C), 131.3 (benzyl 4-C), 130.4 (benzoatyl 1/2-C), 129.4 (benzyl 3-C), 127.2 (benzoatyl 3/4-C), 35.1 (benzyl CH_2); m/z ES+ Found MH^+ 396.9628, $C_{16}H_{10}Cl_2N_2O_2S_s$ requires MH^+ 396.9639.
